# Predictive value of PD-L1 expression in response to immune checkpoint inhibitors for esophageal cancer treatment: A systematic review and meta-analysis

**DOI:** 10.3389/fonc.2022.1021859

**Published:** 2022-12-15

**Authors:** Maryam Noori, Amir-Mohammad Yousefi, Mohammad Reza Zali, Davood Bashash

**Affiliations:** ^1^ Research Committee, School of Medicine, Iran University of Medical Sciences, Tehran, Iran; ^2^ Department of Hematology and Blood Banking, School of Allied Medical Sciences, Shahid Beheshti University of Medical Sciences, Tehran, Iran; ^3^ Gastroenterology and Liver Diseases Research Center, Research Institute for Gastroenterology and Liver Diseases, Shahid Beheshti University of Medical Sciences, Tehran, Iran

**Keywords:** esophageal cancer, ICI, PD-L1, Nivolumab, combined-positive score, tumor proportion score

## Abstract

**Background:**

Programmed death-ligand-1 (PD-L1) molecule is a well-known predictive biomarker for the efficacy of immune checkpoint inhibitors (ICIs) in several cancers. Present systematic review and meta-analysis aimed at investigating the role of PD-L1 in predicting the effectiveness of programmed death-1 (PD-1)/PD-L1 inhibitors in patients suffering from esophageal cancer.

**Methods:**

We searched PubMed, Scopus, Web of Science, and EMBASE databases as of March 25, 2022, for retrieving the potential relevant randomized controlled trials (RCTs). The pooled hazard ratios (HR) and the corresponding 95% confidence intervals (95%CIs) were calculated for the outcomes of overall survival (OS) and progression-free survival (PFS). The primary objective was to investigate the association between PD-1/PD-L1 inhibitors *vs*. control agents and treatment efficacy in terms of OS in patients with esophageal tumor expressing different values of PD-L1 based on combined-positive score (CPS) and tumor proportion score (TPS). The secondary outcome was the pooled risk of PFS.

**Results:**

Eleven studies with a total of 5,418 participants were included. While there was no difference in the OS of CPS<1 patients in the intervention and the control group, patients bearing esophageal tumors with a CPS≥1 (HR 0.65, 0.56-0.74) treated by ICIs showed a significant improvement in OS relative to the control agents. Accordingly, patients with CPS<5 (HR 0.75, 0.58-0.98), CPS≥5 (HR 0.64, 0.53-0.77), CPS<10 (HR 0.86, 0.76-0.98), and CPS≥10 (HR 0.65, 0.56-0.75) had improved OS; however, a significant longer OS was observed in cases who expressed higher values of CPS=10 (p=0.018). In terms of TPS, a significant greater benefit in prolonging the OS came from TPS≥1% PD-L1 expressing tumors in comparison to TPS<1% tumors, suggesting this cut-off as another predictor of PD-1/PD-L1 inhibitors efficacy. Notably, in the subgroup analysis when the cut-off value of CPS=10 or TPS=1% was selected, Nivolumab was the best ICI that improved the survival of PD-L1 positive patients. In patients with negative PD-L1 expression, Toripalimib is the only ICI which could prolong the OS of patients with the cut-off value of CPS=10.

**Conclusion:**

Among patients suffering from esophageal cancer, PD-L1 CPS=10 and TPS=1% expression thresholds seem to be predictive of a lower rate of mortality when PD-1/PD-L1 inhibitors are administrated; however, further large-scale trials are required for confirming the findings of the present study.

## Highlights

- Patients expressing PD-L1 as CPS≥10 significantly take more advantage of ICIs than those with CPS<10.
- With the cut-off value of CPS=10:

Nivolumab and Pembrolizumab were the only ICIs that improved OS of patients with CPS≥10 tumors.
Toripalimib was the only ICI that improved OS of patients with CPS<10 tumors.

- TPS≥1% PD-L1 expressing tumors showed a significant longer OS in comparison to TPS<1% tumors.
- With the cut-off value of TPS=1%:

Nivolumab and Camrelizumab could significantly improve the OS in patients with TPS≥1% tumors.
None of the ICIs were able to longer the OS in patients with TPS<1% tumors.

- Anti-PD-1 therapies are significantly more beneficial for increasing OS than PD-L1 inhibitors.

## 1 Introduction

Esophageal cancer is the seventh most common malignancy in the world and the sixth leading cause of cancer-related death. Currently, chemotherapy remains the standard treatment for first- and second-line management of this malignancy (1–3). In more recent years, immune checkpoint inhibitors (ICIs) have also been actively tested with some encouraging results, especially for patients with tumors that are characterized by a deficiency in mismatch repair enzymes and high microsatellite instability (4–6). However, one of the most important challenges in this area is identifying the patients who would benefit from immunotherapy modalities.

Several predictive biomarkers can be used to identify the patients which clinically respond better to ICIs (7). Since most gastrointestinal cancers overexpress programmed death-ligand 1 (PD-L1), immunohistochemistry (IHC) analysis of this molecule seems to be the most widely validated method for selecting patients for ICI therapy (8); however, the main challenge facing the application of PD-L1 expression as a biomarker is the ambiguity of the relationship between PD-L1 expression and the clinical efficacy of ICIs relative to routine therapies (9). Furthermore, defining a borderline for PD-L1 positivity is still evolving, and the emerging trials have used various PD-L1 expression cut-offs based on combined-positive score (CPS) and tumor proportion score (TPS) (10). Additionally, an optimal setting for immunotherapy of esophageal cancer based on PD-L1 expression status remained unanswered (11).

In the present systematic review and meta-analysis, we sought for identifying the randomized control trials (RCTs) investigating the role of PD-L1 expression in responding to ICIs. In addition, we performed the meta-analysis with the aim of finding a suitable PD-L1 cut-off for improving the clinical efficacy of ICIs based on TPS and CPS.

## 2 Methods

Present systematic review and meta-analysis was conducted in line with the Preferred Reporting Items for Systematic Reviews and Meta-Analyses (PRISMA) guidelines (12).

### 2.1 Search strategy

Eligible RCTs that compared the efficacy of ICIs with control agents based on the expression of PD-L1 were identified through a comprehensive literature search in PubMed, Scopus, Web of Science, and EMBASE databases. We searched the RCTs that were published in English as of March 25, 2022, using the key terms including (“Esophageal Squamous Cell Carcinoma” OR “Esophageal Tumor” OR “Esophageal Cancer” OR “Gastroesophageal Junction Adenocarcinoma”) AND (“PD-L1 Inhibitor” OR “PD-1 Inhibitor” OR “Pembrolizumab” OR “Nivolumab” OR “Durvalumab” OR “Camrelizumab” OR “Atezolizumab”) AND (“Randomised Trial” OR “Clinical Trial” OR “Controlled Clinical Trial”). The detailed information on search strategy is represented in **Table S1**. We also reviewed the published abstracts from annual conferences of the American Society of Clinical Oncology (ASCO), the European Society of Medical Oncology (ESMO), and the American Association for Cancer Research (AACR). In the case where duplicate studies were identified, the most recent and complete version of the data was included.

### 2.2 Study selection

The yield of the search was exported to EndNote software (Clarivate Analytics, Philadelphia, PA, USA). After removing the duplicate records, two authors independently reviewed the title/abstract of the publications according to the inclusion and exclusion criteria. Afterward, the same two authors screened the full-texts of the selected records, independently. Discrepancies were resolved by consulting a third author.

### 2.3 Eligibility criteria

We included RCTs if the following criteria were met: (1) patients with esophageal cancer or gastroesophageal junction adenocarcinoma aged 18 years or older were enrolled; (2) a PD-1/PD-L1 inhibitor was given to the intervention group; (3) placebo, chemotherapy, radiotherapy was given to the control group; and (4) the outcomes of interest (i.e. overall survival [OS] and progression-free survival [PFS]) were reported based on the expression of PD-L1.

The exclusion criteria were as follows: (1) trials including only patients with gastric cancer; (2) trials that administrated ICIs targeting CTLA-4 or other types of ICIs targeting innate immune system to all cohorts of patients in the experimental group; and (3) other types of studies such as editorials, letters to the editor, commentaries, case reports, case series, case-controls, cohorts, cross-sectionals, re-analysis of previously published articles, and any types of review articles.

### 2.4 Data extraction

Two authors independently extracted the following data from included trials using a predefined information sheet. Disagreements were addressed by consensus. We extracted the following items for each included trial: (1) study characteristics including the name of the first author, year of publication or conference presentation, study title, clinical trial identification number, the acronym of the trial, country of origin, and phase of the trial; (2) characteristics of participants including the total number of patients, inclusion and exclusion criteria, age, sex, and type of tumor in both intervention and control groups; (3) intervention and comparison characteristics including type, dose, and schedule of intervention and control medication(s); (4) PD-L1 expression characteristics including the threshold, type of PD-L1 antibody clone, and PD-L1 IHC scoring method; and (5) efficacy measures including OS and PFS.

### 2.5 Quality assessment

Using the Cochrane Collaboration’s risk of bias tool (RoB 2), two independent reviewers assessed the quality of the included papers. This tool examines the risk of bias of RCTs in five domains: randomization process, deviation from intended interventions, missing outcome data, measurement of the outcome, and selection of the reported result. Eventually, the methodological quality of included trials was rated as low risk of bias, some concerns, and high risk of bias.

### 2.6 Data synthesis

The primary objective was to investigate the association between PD-1/PD-L1 inhibitors *vs*. control agents and treatment efficacy in terms of OS in patients with esophageal tumors expressing different values of PD-L1. The secondary outcome was the pooled risk of PFS. The OS and PFS outcomes were measured with hazard ratios (HRs) and the corresponding 95% confidence intervals (CIs) which were extracted from each study. We used Cochrane’s Q statistic to assess between-study heterogeneity and calculated the I-square statistic. A random-effect model was applied if obvious heterogeneity was present (*I* ^2^ >50%), otherwise, a fixed-effect model was chosen (13). The subgroup analysis was conducted according to the type of ICI medication and the molecular target of the ICI agents. The variations in treatment effect between subgroups were assessed using interaction tests. As was recommended by Sterne et al., examination of publication bias using funnel plots was only evaluated if at least ten articles were included in the systematic review (14).

We used STATA version 17.0 (Stata Corporation, College Station, TX, USA) to perform all of the analyses. The risk of bias summary was illustrated using the Risk-of-bias VISualization (robvis), a web application designed to visualize the results of quality assessment of systematic reviews (15). A p-value less than 0.05 would be treated as statistically significant.

## 3 Results

**Figure 1:**
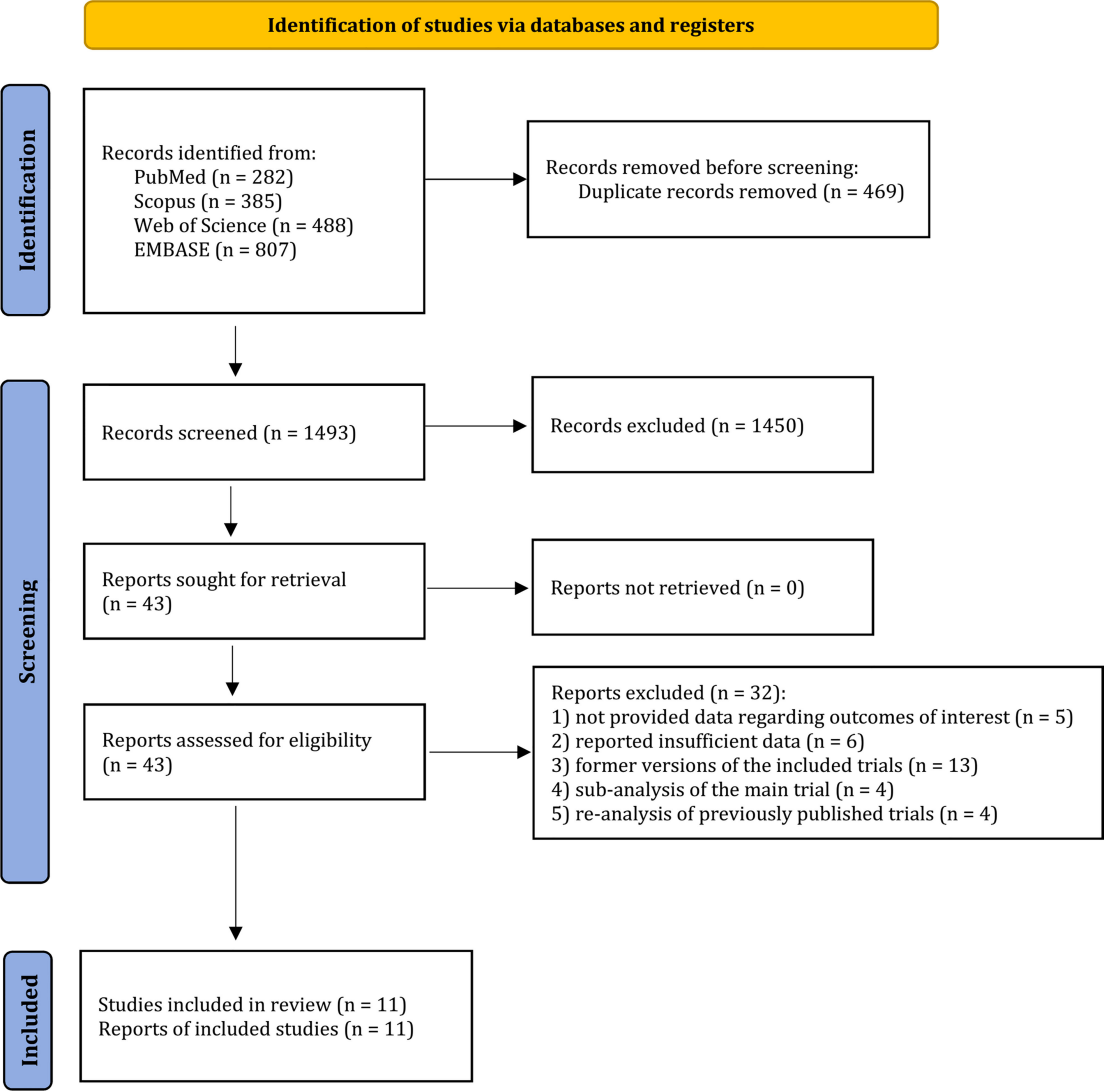
Study selection process of the meta-analysis.

A total of 1,962 studies were retrieved from PubMed (n=282), Scopus (n=385), Web of Science (n=488), and EMBASE (n=807). Of these results, 469 duplicated records were excluded. After title and abstract screening, 1450 studies were not eligible, leaving 43 records for full-texts reviewing. Thereafter, 32 studies did not meet the inclusion and exclusion criteria as a consequence of the following reasons: 1) not provided data regarding outcomes of interest (16–20); 2) reported insufficient data (21–26); 3) former versions of the included trials (27–39); 4) sub-analysis of the main trial (40–43); and 5) re-analysis of previously published trials (44–47). Eventually, 11 potential studies with a total of 5,418 participants were included to the present systematic review and meta-analysis (48–58) (**Figure 1**).

### 3.1 Study characteristics

The detailed characteristics of the included publications are summarized in **Table 1**. All eligible trials were published between 2019 and 2022. Nine studies were in phase III (48–54, 56, 57) and two studies were in phase II (55, 58). Of the eligible trials, six were conducted as double-blinded (52, 54–58) and five as open-label (48–51, 53) fashions. Four trials administered ICIs for the first-line therapy (49, 54, 56, 57), five trials as the second line or more (48, 50, 51, 53, 58), and two trials as the adjuvant therapy (52, 55). Patients with esophageal cancer in the experimental groups were given Nivolumab in three trials (49, 51, 52), Pemberlizumab in three trials (48, 53, 56), Camrelizumab in two trials (50, 54), as well as Durvalumab (55), Toripalimab (57), and Sintilimab (58) each in one trial. Furthermore, ICIs were administrated along with chemotherapy agents for the control group in four studies (49, 54, 56, 57). Regarding PD-L1 immunohistochemistry (IHC) scoring method, four trials used the IHC antibody clone 22C3 (48, 53, 56, 58), three trials used a clone 28-8 antibody (49, 51, 52), two trials used 6E8 clone (50, 54), one trial used SP263 clone (55), and the other trial used JS311 clone (57). Expression of PD-L1 for esophageal tumor was measured based on TPS and CPS which are defined as the ratio of PD-L1 stained tumor cells to the total number of viable tumor cells and the ratio of PD-L1 stained tumor cells and immune cells to the total number of viable tumor cells, respectively. The PD-L1 cut-off values employed by included studies are summarized in **Table 2**.

**Table 1:** Characteristics of the included studies.

First author	Year	NCT identifier	Phase	Trial design	Histology of tumor	Total No. of Pts	Line of therapy	Treatment regimen intervention			Treatment regimen control		PD-L1 IHC scoring method
								Type of ICI	Dose and schedule	Additional medication	Type of control medication	Dose and schedule	
Doki et al.	2022	NCT03143153	3	randomized, open-label	SCC	645	1st	Nivolumab	240 mg Q2W	Chemotherapy	Cisplatin/Fluorouracil	Fluorouracil: 800 mg/m² on days 1 through 5 Cisplatin: 80 mg/m² on day 1	PD-L1 IHC 28-8 pharmDx assay
Huang et al.	2020	NCT03099382	3	randomized, open-label	SCC	457	2nd or more	Camrelizumab	200 mg Q2W	_	Docetaxel/Irinotecan	Docetaxel: 75 mg/m² on day 1 of each 3-week cycle Irinotecan: 180 mg/m² on day 1 of each 2-week cycle	PD-L1 IHC 6E8 pharmDx assay
Kato et al.	2019	NCT02569242	3	randomized, open-label	SCC	419	2nd or more	Nivolumab	240 mg Q2W	_	Paclitaxel/Docetaxel	Paclitaxel: 100 mg/m² QW Docetaxel: 75 mg/m² Q3W	PD-L1 IHC 28-8 pharmDx assay
Kelly et al.	2021	NCT02743494	3	randomized, double-blind	SCC and adenocarcinoma	794	Adjuvant	Nivolumab	240 mg Q2W for 16 weeks, followed by a dose of 480 mg Q4W	_	Placebo	_	PD-L1 IHC 28-8 pharmDx assay
Sun et al.	2021	NCT03189719	3	randomized, double-blind	SCC and adenocarcinoma	749	1st	Pembrolizumab	200 Q3W	Chemotherapy	Fluorouracil/Cisplatin	Fluorouracil: 800 mg/m² on days 1–5 Cisplatin: 80 mg/m² on day 1	PD-L1 IHC 22C3 pharmDx assay
Cao et al.	2022	NCT02564263	3	randomized, open-label	SCC and adenocarcinoma	340	2nd or more	Pembrolizumab	200 Q3W	_	Paclitaxel/Docetaxel/Irinotecan	Paclitaxel: 80-100 mg/m² on days 1, 8, and 15 of Q4W Docetaxel: 75 mg/m² on day 1 of Q3W Irinotecan: 180 mg/m² on day 1 of Q2W	PD-L1 IHC 22C3 pharmDx assay
Kojima et al.	2020	NCT02564263	3	randomized, open-label	SCC and adenocarcinoma	628	2nd or more	Pembrolizumab	200 Q3W	_	Paclitaxel/Docetaxel/Irinotecan	Paclitaxel: 80-100 mg/m² on days 1, 8, and 15 of Q4W Docetaxel: 75 mg/m² on day 1 of Q3W Irinotecan: 180 mg/m² on day 1 of Q2W	PD-L1 IHC 22C3 pharmDx assay
Luo et al.	2021	NCT03691090	3	randomized, double-blind	SCC	596	1st	Camrelizumab	200 Q3W	Chemotherapy	Paclitaxel/Cisplatin	Paclitaxel: 175 mg/m² on day 1 of Q3W Cisplatin: 75 mg/m² on day 1 of Q3W	PD-L1 IHC 6E8 pharmDx assay
Park et al.	2022	NCT02520453	2	randomized, double-blind	SCC	86	Adjuvant	Durvalumab	20 mg/kg Q4W	_	Placebo	_	PD-L1 IHC SP263 pharmDx assay
Wang et al.	2022	NCT03829969	3	randomized, double-blind	SCC	514	1st	Toripalimab	240 mg Q3W	Chemotherapy	Paclitaxel/Cisplatin	Paclitaxel: 175 mg/m² on day 1 of Q3W Cisplatin: 75 mg/m² on day 1 of Q3W	PD-L1 IHC JS311 pharmDx assay
Xu et al.	2022	NCT03116152	2	randomized, double-blind	SCC	190	2nd or more	Sintilimab	200 mg Q3W	_	Paclitaxel/Irinotecan	Paclitaxel: 175 mg/m² Q3W Irinotecan: 180 mg/m² Q2W	PD-L1 IHC 22C3 pharmDx assay

ICI, immune checkpoint inhibitors; SCC, Squamous-cell carcinoma; PD-L1, Programmed death-ligand 1; IHC, Immunohistochemistry; QW, Every week; Q2W, every 2 weeks; Q3W, Every 3 weeks; Q4W, Every 4 weeks; Pts, Patients; No.: Number.

**Table 2:** PD-L1 cut-off values employed by included studies.

First author	OS	PFS
Doki et al.	CPS=1, 5, 10 TPS= 1%, 5%, 10%	TPS= 1%
Huang et al.	TPS= 1%, 5%, 10%	TPS= 1%, 5%, 10%
Kato et al.	TPS= 1%, 5%, 10%	–
Kelly et al.	–	CPS= 5 TPS= 1%
Sun et al.	CPS=10	CPS= 10
Cao et al.	CPS=1, 5	CPS=1, 5, 10
Kojima et al.	CPS=10	CPS= 10
Luo et al.	TPS= 1%, 5%, 10%	TPS= 1%, 5%, 10%
Park et al.	TPS= 1%	TPS= 1%
Wang et al.	CPS=1, 10	CPS=1, 10
Xu et al.	CPS=1, 10 TPS= 1%, 10%	CPS=1, 10 TPS= 1%, 10%

### 3.2 Risk of bias assessment

All of our included studies showed a high risk of bias in RoB2 quality assessment tool. The main domain affecting the quality was bias due to missing outcome data as a result of time-to-event analyses where the censored patients may have caused missingness in the outcome. None of our studies were subjected to bias in terms of the measurement of the outcomes and selection of reported results. The summary of quality assessment results is depicted in **Figure S1**.

### 3.3 Efficacy of PD-1/PD-L1 inhibitors based on PD-L1 expression status by CPS

#### 3.3.1 OS

**Figure 2:**
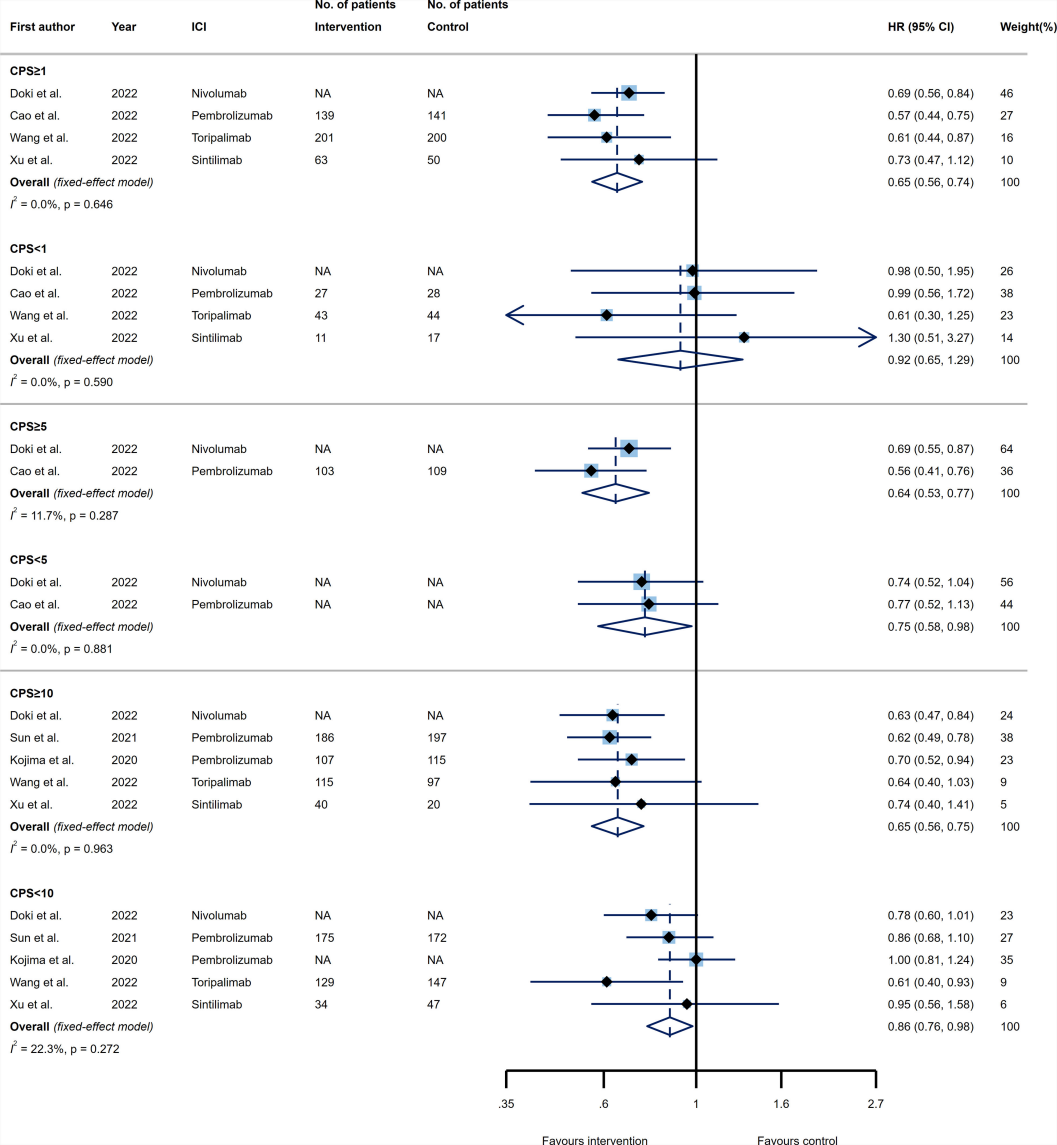
Forest plots of overall survival (OS) in PD-L1 high expression group *vs*. PD-L1 low expression group for the thresholds of combined positive score (CPS)=1 (upper panel), CPS=5 (middle panel), and CPS=10 (lower panel). The squares indicate weight of each study based on the fixed or random‐effect model. The vertical dashed line indicates the overall pooled estimate and the diamond the 95% confidence interval around that pooled estimate. The forest plot was generated using STATA 17.0 (STATA Corp, LLC, TX).

The included RCTs evaluated the efficacy of PD-1/PD-L1 inhibitors in three CPS thresholds of 1, 5, and 10. Comparing the efficacy of ICIs relative to the control agents in terms of OS, four trials evaluated the predictive role of PD-L1 expression through the setting of CPS=1 as the cut-off value. Esophageal tumors with a CPS≥1 treated by ICIs showed a significant improvement in OS (HR 0.65, 95% CI 0.56-0.74), while tumors with a CPS<1 could not benefit from ICIs (HR 0.92, 95% CI 0.65-1.29) as compared to the control treatment. However, no significant difference was detected in reducing the risk of death for patients that had CPS≥1 relative to CPS<1 tumors (p_interaction_= 0.114) (**Figure 2**, upper panel).

Considering the CPS threshold of 5, two trials evaluated the OS of patients with esophageal tumors receiving ICIs based on PD-L1 expression status. Both CPS≥5 and CPS<5 PD-L1-expressing tumors were able to significantly longer the OS of patients (HR 0.64, 95% CI 0.53-0.77 and HR 0.75, 95% CI 0.58-0.98, respectively). In this case, while the OS was increased to a greater extent in patients with CPS≥5 than those with CPS<5, the difference was not statistically significant (p_interaction_= 0.423) (**Figure 2**, middle panel).

Besides, five trials were included for examining the OS according to the CPS threshold of 10. The death rate decreased substantially for either esophageal tumors with CPS≥10 (HR 0.65, 95% CI 0.56-0.75) or CPS<10 (HR 0.86, 95% CI 0.76-0.98) when a PD-1/PD-L1 inhibitor was administrated versus the control group. Interestingly, patients expressing PD-L1 as CPS≥10 took more advantage of PD-1/PD-L1 blockade therapies in terms of OS than patients bearing esophageal tumors with CPS<10 (p_interaction_=0.018) (**Figure 2**, lower panel). It is notable that neither analysis resulted in a remarkable heterogeneity (**Figure 2**).

#### 3.3.2 PFS

**Figure 3:**
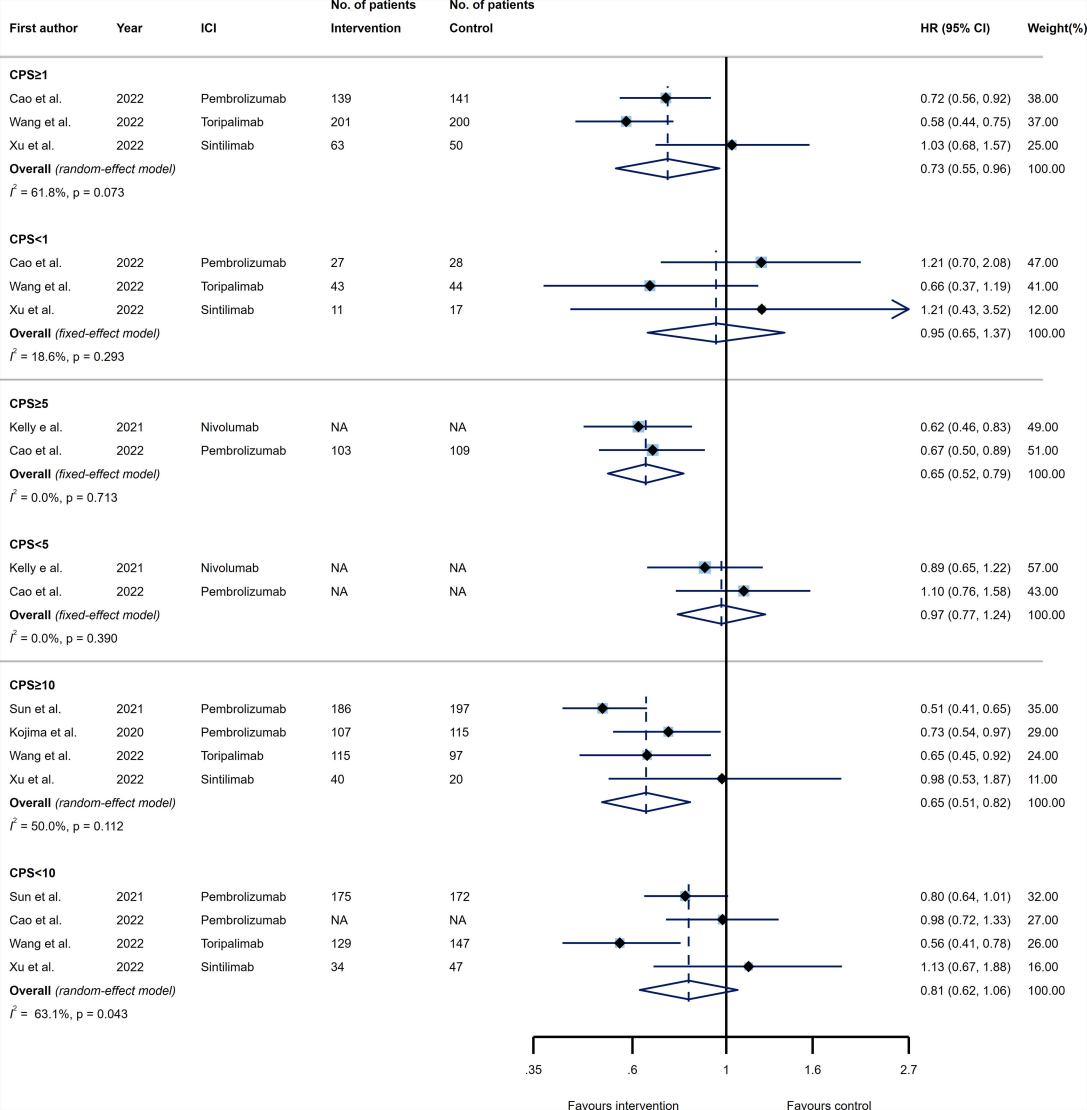
Forest plots of progression-free survival (PFS) in PD-L1 high expression group *vs*. PD-L1 low expression group for the thresholds of combined positive score (CPS)=1 (upper panel), CPS=5 (middle panel), and CPS=10 (lower panel). The squares indicate weight of each study based on the fixed or random‐effect model. The vertical dashed line indicates the overall pooled estimate and the diamond the 95% confidence interval around that pooled estimate. The forest plot was generated using STATA 17.0 (STATA Corp, LLC, TX).

In the next step, we evaluated the predictive effect of PD-L1 expression by CPS over the efficacy of PD-1/PD-L1 inhibitors in terms of PFS. Overall, three, two, and four trials estimating the PFS were included for CPS threshold of 1, 5, and 10, respectively. When comparing the efficacy of PD-1/PD-L1 inhibitors with the agents prescribed for the control group, patients who had CPS≥1, CPS≥5, and CPS≥10 represented substantially longer PFS (HR 0.73, 95% CI 0.55-0.96; HR 0.65, 95% CI 0.52-0.79; and HR 0.65, 95% CI 0.51-0.82, respectively). On the other hand, patients with PD-L1 expression values of CPS<1, CPS<5, and CPS<10 could not benefit from ICIs relative to the control agents (HR 0.95, 95% CI 0.65-1.37; HR 0.97, 95% CI 0.77-1.24; and HR 0.81, 95% CI 0.62-1.06, respectively). Moreover, none of the CPS thresholds were considered predictive for PFS of patients receiving ICIs as compared to the controls, since tumors with higher PD-L1 expression did not decrease the risk of disease progression significantly as compared to the lower values (p_interaction_=0.392, p_interaction_=0.125, and p_interaction_=0.283 for CPS thresholds of 1, 5, and 10, respectively). Evidence of considerable heterogeneity was noted among trials included to the efficacy analysis for CPS≥1 (*I* ^2 =^ 61.8%), CPS≥10 (*I* ^2 =^ 50.0%), and CPS<10 (*I* ^2 =^ 63.1%) (**Figure 3**).

### 3.4 Efficacy of PD-1/PD-L1 inhibitors based on PD-L1 expression status by TPS

#### 3.4.1 OS

**Figure 4:**
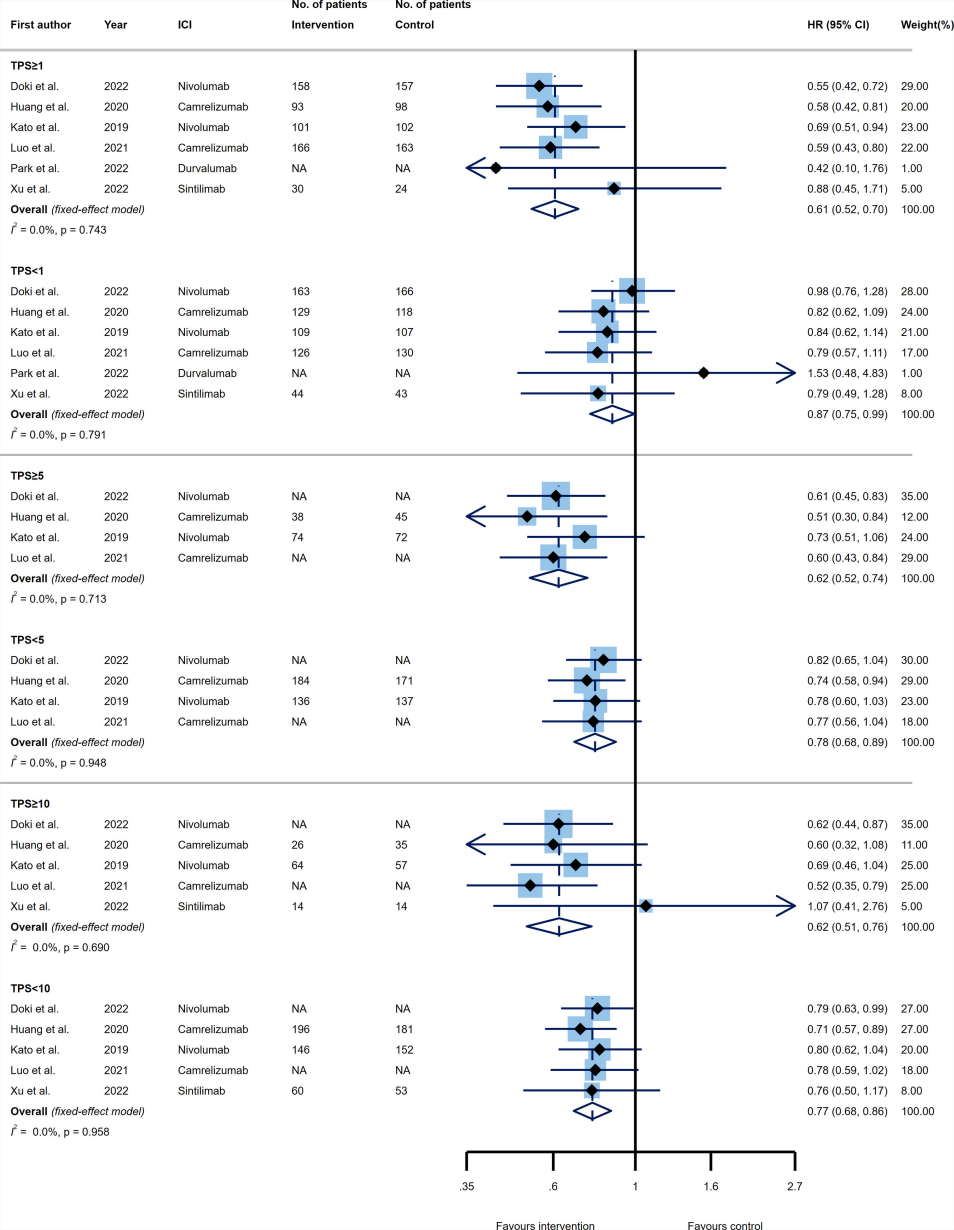
Forest plots of overall survival (OS) in PD-L1 high expression group *vs*. PD-L1 low expression group for the thresholds of tumor proportion score (TPS)=1% (upper panel), TPS=5% (middle panel), and TPS=10% (lower panel). The squares indicate weight of each study based on the fixed or random‐effect model. The vertical dashed line indicates the overall pooled estimate and the diamond the 95% confidence interval around that pooled estimate. The forest plot was generated using STATA 17.0 (STATA Corp, LLC, TX).

Three thresholds of TPS, known as 1%, 5%, and 10% were evaluated as predictive PD-L1 expression cut-offs among our included trials. Six trials examined the OS of patients with esophageal cancer receiving PD-1 or PD-L1 inhibitors versus control agents in two subgroups of patients expressing PD-L1 as TPS≥1% and TPS<1%. Both categories of tumors with TPS≥1% and TPS<1% showed significantly better OS favored ICI (HR 0.61, 95% CI 0.52-0.70 and HR 0.87, 95% CI 0.75-0.99); however, a greater benefit in prolonging the OS came from TPS≥1% PD-L1 expressing tumors. Excitingly, the TPS=1% was another predictor of improved efficacy for PD-1/PD-L1 inhibitors as compared to the control group, where the TPS≥1% tumors versus TPS<1% tumors represented a significantly reduced risk of mortality (p_interaction_=0.006) (**Figure 4**, upper panel).

Considering the TPS=5% cut-off, four RCTs were included. Again the upper (i.e. TPS≥5%) and lower (i.e. TPS<5%) values had a remarkable impact toward increasing the OS of patients receiving ICIs relative to the control group (HR 0.62, 95% CI 0.52-0.74 and HR 0.78, 95% CI 0.68-0.89, respectively), with TPS≥5% indicated larger effect. Of note, the difference between the effect sizes of the two groups did not reach statistically significance, implying that this threshold could not be accounted as a predictive marker for OS (p_interaction_=0.092) (**Figure 4**, middle panel).

Lastly, five trials reported the efficacy of ICIs compared to the control agents base on PD-L1 expressing threshold of TPS=10%. Regarding the OS efficacy analysis, the same pattern followed the estimation of TPS=10%, suggesting that PD-1/PD-L1 inhibitors had a significant impact on improving OS of patients with esophageal cancer for both TPS≥10% (HR 0.62, 95% CI 0.51-0.76) and TPS<10% (HR 0.77, 95% CI 0.68-0.86) tumors; such that the superior impact of TPS≥10% group in reducing the mortality rate did not reveal a substantial difference with TPS<10% group (p_interaction_=0.121) (**Figure 4**, lower panel). Furthermore, none of our analyses had a remarkable between study heterogeneity (**Figure 4**).

#### 3.4.2 PFS

**Figure 5:**
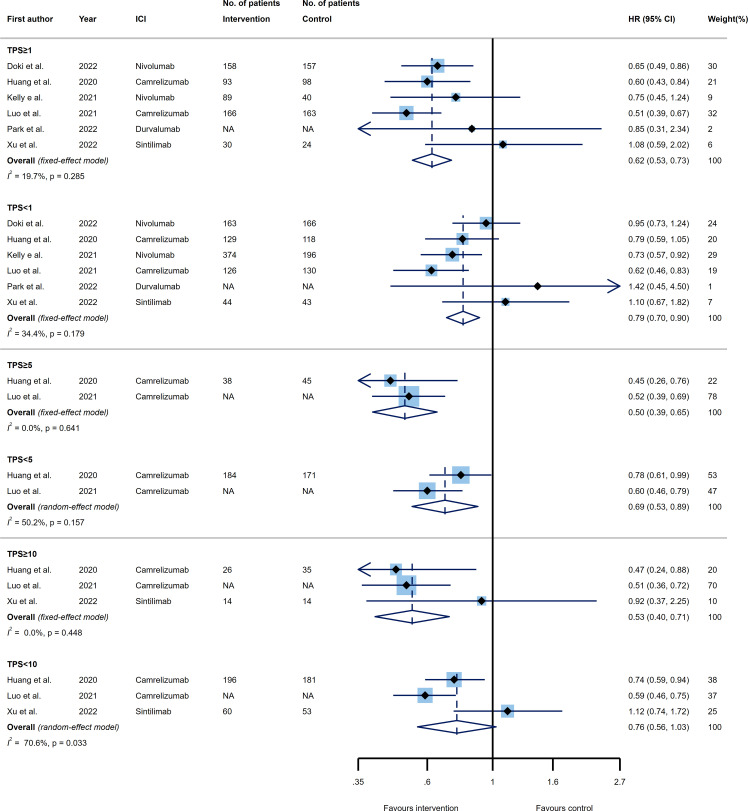
Forest plots of progression-free survival (PFS) in PD-L1 high expression group *vs*. PD-L1 low expression group for the thresholds of tumor proportion score (TPS)=1% (upper panel), TPS=5% (middle panel), and TPS=10% (lower panel). The squares indicate weight of each study based on the fixed or random‐effect model. The vertical dashed line indicates the overall pooled estimate and the diamond the 95% confidence interval around that pooled estimate. The forest plot was generated using STATA 17.0 (STATA Corp, LLC, TX).

In terms of PFS, six, two, and three RCTs assessed the efficacy of PD-1/PD-L1 inhibitors versus control agents according to the PD-L1 expression status by TPS. The pooled estimates showed that the immunotherapeutic modalities targeting PD-1 or PD-L1, reduced the rate of disease progression for higher and lower values of TPS=1% and TPS=5% thresholds (TPS≥1%: HR 0.62, 95% CI 0.53-0.73; TPS<1%: HR 0.79, 95% CI 0.70-0.90; TPS≥5%: HR 0.50, 95% CI 0.39-0.65; TPS<5%: HR 0.69, 95% CI 0.53-0.89). However, the tumors with TPS≥10% demonstrated a significant improvement in PFS (HR 0.53, 95% CI 0.40-0.71) in contrast to the tumors with TPS<10% (HR 0.76, 95% CI 0.56-1.03) that showed no taking advantage from ICIs. Comparing the higher and lower values of each threshold, none of the TPS=1%, TPS=5%, and TPS=10% were able to predict the impact of ICIs on PFS as compared to the control agents (p_interaction_=0.100, p_interaction_=0.224, and p_interaction_=0.283, respectively). The only analyses with a high degree of heterogeneity were TPS<5% (*I* ^2 =^ 50.2%) and TPS<10% (*I* ^2 =^ 70.6%) (**Figure 5**).

### 3.5 Subgroup analysis

#### 3.5.1 CPS=10

We selected the cut-off value of CPS=10 in order to examine whether the type of ICIs is responsible for better response in PD-L1 positive (i.e. CPS≥10) or negative (i.e. CPS<10) tumors. Among PD-L1 positive tumors, Nivolumab (HR 0.63, 95% CI 0.47-0.84) and Pembrolizumab (HR 0.65, 95% CI 0.54-0.78) were the only ICIs that improved the survival of the affected patients considerably, as compared to the control group. On the other hand, patients with PD-L1 negative tumors could only take advantage of Taripalimib (HR 0.61, 95% CI 0.40-0.93) in prolonging the survival time relative to the control medications (**Figure S2**).

#### 3.5.2 TPS=1%

Next, we examined the effect of different ICIs in OS of patients affected by esophageal PD-L1 positive (i.e. TPS≥1%) and PD-L1 negative (i.e. TPS<1) tumors with the cut-off value of TPS=1%. The pooled estimate revealed that in PD-L1 positive patients, Nivolumab (HR 0.61, 95% CI 0.50-0.74) and Camrelizumab (HR 0.59, 95% CI 0.47-0.73) could significantly improve the OS as compared to the control group. However, none of the ICIs were able to longer the OS in PD-L1 negative patients suffering from the esophageal cancer. Regarding the cellular target of ICIs, both PD-L1 positive and negative patients receiving PD-1 blockade therapies showed a decreased risk of mortality (HR 0.61, 95% CI 0.53-0.70 and HR 0.89, 95% CI 0.75-0.99, respectively), while PD-L1 blockade therapies had no effect on OS versus the control agents for both groups of PD-L1 expression (**Figure S3**).

## 4 Discussion

Immunotherapy has revolutionized the treatment of multiple cancers in recent years. ICIs are among these promising treatments that have potentially improved the survival of patients with different types of malignancies. However, it is still a major question for clinicians that which patients may benefit more from prescription of ICIs. Regarding the high rate of immune-related adverse events as well as the high price of these kinds of medications, finding patients who are more likely to respond to ICIs would be an important issue. According to several publications, PD-L1 expression may become a potential candidate for predicting the subsequent clinical response to ICIs (7, 8, 59, 60); however, a number of challenges have been found in this way. For instance, while most of the studies found a positive correlation between the expression of PD-L1 and response to ICIs and it has been proved that PD-L1 positive tumors are more likely to respond to immunotherapy, some investigations reported a considerable number of patients with PD-L1 negative tumors which may also respond to ICIs (61, 62). Accordingly, a recent meta-analysis of six RCTs has demonstrated that PD-L1 expression did not affect the OS and PFS of patients with non-small cell lung cancer (NSCLC) receiving ICIs as compared to the control agents, implying a constant positive effect of ICIs over control group regardless of PD-L1 expression level (60). It could be assumed that the type of tumor or in part the outcome measure may also play a role in the predictive accuracy of PD-L1 expression. In this case, it has been reported that PD-L1 expression was predictive of response for patients with non-squamous NSCLC (63); however, the response benefit of ICIs was independent of PD-L1 expression for squamous NSCLC (64). As another challenge, several studies reported that PD-L1 is an unreliable biomarker owing to its dynamic changes and the expression of this biomarker can be increased by several factors such as exposure to immunotherapy (65). Furthermore, there is still no general consensus regarding the diagnostic assays for measuring PD-L1 expression, with main inconsistencies about which antibody to use, which cells to stain, and what cut-off value to choose (8, 59).

Based on our results, although patients suffering from esophageal cancer with a CPS<1 could not benefit from ICIs compared to the control agents, those who had CPS≥1, CPS<5, CPS≥5, CPS<10, and CPS≥10 showed a significant improvement in OS. Notably, while no significant difference was detected in reducing the risk of death according to the CPS threshold of 1 and 5, patients expressing PD-L1 as CPS≥10 have significantly taken more advantage of PD-1/PD-L1 blockade therapies than patients bearing esophageal tumors with CPS<10. In accordance with our results, Shieh et al. reported that advanced cervical cancer patients with PD-L1 CPS≥10 demonstrated a statistically higher response rate to ICIs than those with CPS<10 (66). Moreover, analyses of recent clinical trials in patients with gastric or gastroesophageal junction cancer showed that patients with CPS≥10 may derive greater benefit from pembrolizumab in terms of OS (37, 67).

As a result, we selected the cut-off value of CPS=10 in order to examine whether the type of ICIs is responsible for better response in PD-L1 positive (i.e. CPS≥10) or negative (i.e. CPS<10) tumors. Among PD-L1 positive tumors, two well-known anti-PD1 inhibitors, Nivolumab and Pembrolizumab, were the only ICIs that improved the survival of the affected patients considerably as compared to the control group. In line, a recent analysis survey showed consistent improvements toward more favorable clinical outcomes with pembrolizumab across lines of therapy in gastroesophageal cancer patients with CPS≥10 (68); similar results were reported for Nivolumab (69, 70). Notably, patients with PD-L1 negative tumors could only take advantage of Toripalimib which significantly prolonged the survival time relative to the control medications; the reason for this apposite response to Toripalimib is not clearly understood and deserve more research.

In addition to CPS, we also assessed the efficacy of ICIs in three TPS thresholds of 1%, 5%, and 10%. While both higher and lower values of TPS=1% demonstrated significantly better OS for ICIs compared to the control, a significantly greater benefit in prolonging the OS came from TPS≥1% PD-L1 expressing tumors in comparison to TPS<1% tumors, suggesting another predictor of improved efficacy for PD-1/PD-L1 inhibitors. In accordance with our findings, Zou and colleagues reported that the rate of objective response was 7% higher in breast cancer patients expressing PD-L1 as TPS≥1% compared with TPS<1% after receiving ICIs (71). In addition, the higher values of PD-L1 TPS showed longer OS relative to the lower values (i.e. TPS<1%) in patients with NSCLC (72).

By investigating the effect of different ICIs in OS of patients with the cut-off value of TPS=1% we found that Nivolumab and Camrelizumab could significantly improve the OS in PD-L1 positive (i.e. TPS≥1%) patients as compared to the control group, whereas none of the ICIs were able to longer the OS in PD-L1 negative patients (i.e. TPS<1%). In line, in an analysis of patients with NSCLC who were on Camrelizumab, it was revealed that the objective response rate was improved with increasing the PD-L1 TPS level (73). Likewise, the NSCLC patients treated with Nivolumab monotherapy reached higher levels of disease control rate when they expressed PD-L1 as TPS≥1% compared to TPS<1% (74). Notably, while PD1 inhibitors showed a decreased risk of mortality in both PD-L1 positive and negative patients, PD-L1 blockades had no significant effect on OS in both groups of PD-L1 expression when compared with the control group. Similarly, it has been demonstrated that anti-PD-1 therapies were significantly more beneficial for increasing OS and PFS than PD-L1 inhibitors in advanced esophageal cancer (75). Altogether, our results could have implications for clinicians when they are trying to make a decision on whether patients can take advantage of ICI therapy. In this case, long-term management schedules could be planned based on the predicted survival rates.

To the best of our knowledge, present study is the first meta-analysis evaluating the predictive effect of PD-L1 expression in esophageal cancer. While prior meta-analysis confirmed the predictive effect of PD-L1 expression in NSCLC and renal cell carcinoma (60, 76), an examination for finding the suitable threshold was not established. Despite the comprehensive nature of the systematic review undertaken, our study has some potential drawbacks. First, we observed a high degree of heterogeneity across some of the pooled analyses; we believe that the heterogeneity was mainly due to the differences in the lines of therapy, varying follow-up durations, and many other factors among these studies. Second, although we enrolled the most up-to-dated clinical trials across databases, the validity of our study was based on the quality of the reviewed trials and some types of biases that originated from the nature of trials may affect the generalizability of the overall findings. Third, our study was performed at the trial level instead of the individual level, and as a result, a group of patients with poor performance status are missed in data interpretation; thus, the survival benefit and predictive value of PD-L1 in a real-world population with comorbidities and poor performance status could be lower. Forth, results of some ongoing trials, such as NCT02352948, NCT02581943, NCT02409342, NCT02273375, and NCT03091491, have not yet been published and hence inclusion of these trials in the future meta-analyses may alter the overall results. Finally, and as the last limitation, the findings should be interpreted with caution due to a relatively small number of included studies and obviously, further investigations are required to confirm our results in a larger variety of clinical trials. Also, the validity of our results could be measured in *post-hoc* analysis of currently published trials with examining all PD-L1 TPS and CPS thresholds.

## 5 Conclusion

The results of our study demonstrated that the cut-off values of CPS=10 and TPS=1% were the most proper borderlines to determining PD-L1 positivity; indeed, patients expressing PD-L1 as CPS≥10 or TPS≥1% took more advantage of ICIs in comparison to PD-L1 negative patients (i.e., CPS<10 or TPS<1%). Interestingly, when the cut-off value of CPS=10 or TPS=1% was selected, we found that Nivolumab was the best ICIs that improved the survival of PD-L1 positive patients (CPS≥10 and TPS ≥1%). Notably, while none of the ICIs could improve the survival of patients with PD-L1 TPS<1%, Toripalimib was the only ICI which could prolong the OS of patients with CPS<10. Taken together, this meta-analysis emphasis on the usefulness of PD-L1 expression as a potential predictive biomarker to select treatment in esophageal cancer. However, there are still many uncertainties on this subject and the efficacy of the aforementioned predictive cut-off values and suggested ICIs should be further explored in clinical studies for all other cancers.

## Data availability statement

The original contributions presented in the study are included in the article/**Supplementary Material**. Further inquiries can be directed to the corresponding author.

## Author contributions

MN and DB designed the study. MN and A-MY performed the study selection and data extraction. MN conducted the analysis. MN, A-MY, and DB wrote the first draft of the manuscript. MN, A-MY, MZ, and DB critically revised the manuscript. All authors reviewed the drafted manuscript for critical content. All authors contributed to the article and approved the submitted version.

## References

[1] Bray F Ferlay J Soerjomataram I Siegel LR Torre AL Ahmedin D . V. m. GLOBOCAN estimates of incidence and mortality worldwide for 36 cancers in 185 countries. Global Cancer Stat (2018) 70(4):313. doi: 10.3322/caac.21492

[2] Al-Batran S-E Hartmann JT Probst S Schmalenberg H Hollerbach S Hofheinz R . Phase III trial in metastatic gastroesophageal adenocarcinoma with fluorouracil, leucovorin plus either oxaliplatin or cisplatin: A study of the arbeitsgemeinschaft internistische onkologie. J Clin Oncol (2008) 26(9):1435–42. doi: 10.1200/JCO.2007.13.9378 18349393

[3] Bouché O Raoul JL Bonnetain F Giovannini M Etienne PL Lledo G . Randomized multicenter phase II trial of a biweekly regimen of fluorouracil and leucovorin (LV5FU2), LV5FU2 plus cisplatin, or LV5FU2 plus irinotecan in patients with previously untreated metastatic gastric cancer: A federation francophone de cancerologie digestive group study–FFCD 9803. J Clin Oncol (2004) 22(21):4319–28. doi: 10.1200/JCO.2004.01.140 15514373

[4] Le DT Durham JN Smith KN Wang H Bartlett BR Aulakh LK . Mismatch repair deficiency predicts response of solid tumors to PD-1 blockade. Science (2017) 357(6349):409–13. doi: 10.1126/science.aan6733 PMC5576142 28596308

[5] Fuchs CS Doi T Jang RW Muro K Satoh T Machado M . Safety and efficacy of pembrolizumab monotherapy in patients with previously treated advanced gastric and gastroesophageal junction cancer: Phase 2 clinical KEYNOTE-059 trial. JAMA Oncol (2018) 4(5):e180013. doi: 10.1001/jamaoncol.2018.0013 29543932 PMC5885175

[6] Kitagawa Y Doki Y Kato K Ura T Kojima T Tsushima T . Two year survival and safety update for esophageal squamous cell carcinoma treated with nivolumab (ATTRACTION-01/ONO-4538-07). Ann Oncol (2017) 28:v218. doi: 10.1093/annonc/mdx369.022

[7] Uruga H Mino-Kenudson M . Predictive biomarkers for response to immune checkpoint inhibitors in lung cancer: PD-L1 and beyond. Virchows Archiv (2021) 478(1):31–44. doi: 10.1007/s00428-021-03030-8 33486574

[8] Doroshow DB Bhalla S Beasley MB Sholl LM Kerr KM Gnjatic S . PD-L1 as a biomarker of response to immune-checkpoint inhibitors. Nat Rev Clin Oncol (2021) 18(6):345–62. doi: 10.1038/s41571-021-00473-5 33580222

[9] Teng F Meng X Kong L Yu J . Progress and challenges of predictive biomarkers of anti PD-1/PD-L1 immunotherapy: A systematic review. Cancer Lett (2018) 414:166–73. doi: 10.1016/j.canlet.2017.11.014 29155348

[10] Huang J Teng X . Expression of PD-L1 for predicting response to immune checkpoint inhibitors in metastatic urothelial carcinoma: A systematic review and meta-analysis. Curr Oncol (2020) 27(6):656–63. doi: 10.3747/co.27.6437 PMC7755433 33380881

[11] Jackie Oh S Han S Lee W Lockhart AC . Emerging immunotherapy for the treatment of esophageal cancer. Expert Opin Investigational Drugs (2016) 25(6):667–77. doi: 10.1517/13543784.2016.1163336 26950826

[12] Page MJ McKenzie JE Bossuyt PM Boutron I Hoffmann TC Mulrow CD . The PRISMA 2020 statement: an updated guideline for reporting systematic reviews. BMJ (2021) 372:n71. doi: 10.1136/bmj.n71 33782057 PMC8005924

[13] Higgins JP Thomas J Chandler J Cumpston M Li T Page MJ . Cochrane handbook for systematic reviews of interventions. Wiley Online library (2019). 10.1002/14651858.ED000142 PMC10284251 31643080

[14] Sterne JAC Sutton AJ Ioannidis JPA Terrin N Jones DR Lau J . Recommendations for examining and interpreting funnel plot asymmetry in meta-analyses of randomised controlled trials. BMJ (2011) 343:d4002. doi: 10.1136/bmj.d4002 21784880

[15] McGuinness LA Higgins JPT . Risk-of-bias VISualization (robvis): An r package and shiny web app for visualizing risk-of-bias assessments. Res Synthesis Methods (2020) 12(1):55–61. doi: 10.1002/jrsm.1411 32336025

[16] Guimbaud R Quero L Vendrely V Tougeron D Benerezy K Samalin E . PRODIGE67_UCGI33 ARION: Association of radiochemotherapy and immunotherapy for the treatment of unresectable oesophageal cancer: A comparative randomized phase II trial. Ann Oncol (2019) 30:v322. doi: 10.1093/annonc/mdz247.163

[17] Ko AH Lee J Alsina M Ajani JA Bang YJ Chung HC . Phase Ib/II open-label, randomized evaluation of 2L atezolizumab (atezo) + PEGPH20 versus control in MORPHEUSpancreatic ductal adenocarcinoma (M-PDAC) and MORPHEUS-gastric cancer (MGC). J Clin Oncol (2020) 38(15):4540. doi: 10.1200/JCO.2020.38.15_suppl.4540

[18] Liu N Liu Z Zhou Y Niu Z Jiang H Zhu Y . Efficacy and safety of camrelizumab combined with FLOT versus FLOT alone as neoadjuvant therapy in patients with resectable locally advanced gastric and gastroesophageal junction adenocarcinoma who received D2 radical gastrectomy. J Clin Oncol (2021) 39(15 SUPPL):e16020. doi: 10.1200/JCO.2021.39.15_suppl.e16020

[19] Oh DY Ajani JA Bang YJ Chung HC Lacy J Lee J . Phase Ib/II open-label, randomized evaluation of 2L atezolizumab (atezo) + BL-8040 versus control in MORPHEUSpancreatic ductal adenocarcinoma (M-PDAC) and MORPHEUS-gastric cancer (MGC). J Clin Oncol (2020) 38(4):712. doi: 10.1200/JCO.2020.38.4_suppl.712

[20] Pauligk C Götze TO Thuss-Patience PC Riera-Knorrenschild J Goekkurt E Ettrich TJ . Modified FOLFOX versus modified FOLFOX plus nivolumab and ipilimumab in patients with previously untreated advanced or metastatic adenocarcinoma of the stomach or gastroesophageal junction – safety results from AIO-STO-0417: A randomized phase II trial of the German gastric group of the AIO. Ann Oncol (2020) 31:S908. doi: 10.1016/j.annonc.2020.08.1949

[21] Ajani J El Hajbi F Cunningham D Alsina M Thuss-Patience P Scagliotti G . O-15 randomized, phase 3 study of second-line tislelizumab vs chemotherapy in advanced or metastatic esophageal squamous cell carcinoma (RATIONALE 302) in the overall population and Europe/North America subgroup. Ann Oncol (2021) 32:S225. doi: 10.1016/j.annonc.2021.05.807

[22] Choi YY Kim H Shin SJ Kim H Lee J Yang HK . Microsatellite instability and programmed cell death-ligand 1 expression in stage II/III gastric cancer *Post hoc* analysis of the CLASSIC randomized controlled study. Ann Surg (2019) 270(2):309–16. doi: 10.1097/SLA.0000000000002803 29727332

[23] Janjigian YY Kawazoe A Yañez P Li N Lonardi S Kolesnik O . The KEYNOTE-811 trial of dual PD-1 and HER2 blockade in HER2-positive gastric cancer. Nature (2021) 600(7890):727–30. doi: 10.1038/s41586-021-04161-3 PMC8959470 34912120

[24] Kang YK Satoh T Ryu MH Chao Y Kato K Chung HC . Nivolumab (ONO-4538/BMS-936558) as salvage treatment after second or later-line chemotherapy for advanced gastric or gastroesophageal junction cancer (AGC): A double-blinded, randomized, phase III trial. J Clin Oncol (2017) 35(4):2. doi: 10.1200/JCO.2017.35.4_suppl.2

[25] Okada M Kato K Cho BC Takahashi M Lin CY Chin K . Three-year follow-up and response-survival relationship of nivolumab in previously treated patients with advanced esophageal squamous cell carcinoma (ATTRACTION-3). Clin Cancer Res (2022) 28(15):3277–86. doi: 10.1158/1078-0432.ccr-21-0985 PMC9662935 35294546

[26] Wei XL Xu N Shen L Dai G Yuan X Chen Y . Clinical response and biomarker analysis of a phase II basket trial of toripalimab, a PD-1 mAb in combination with standard chemotherapy as a first-line treatment for patients with solid tumors. chao ren. J Clin Oncol (2020) 38(15):e15083. doi: 10.1200/JCO.2020.38.15_suppl.e15083

[27] Boku N Kang YK Satoh T Chao Y Kato K Chung HC . A phase 3 study of nivolumab (Nivo) in previously treated advanced gastric or gastroesophageal junction (G/GEJ) cancer: Updated results and subset analysis by PD-L1 expression (ATTRACTION-02). Ann Oncol (2017) 28:v209. doi: 10.1093/annonc/mdx369.001

[28] Chen LT Satoh T Ryu MH Chao Y Kato K Chung HC . A phase 3 study of nivolumab in previously treated advanced gastric or gastroesophageal junction cancer (ATTRACTION-2): 2-year update data. Gastric Cancer (2020) 23(3):510–9. doi: 10.1007/s10120-019-01034-7 PMC7165140 31863227

[29] Cho BC Kato K Takahashi M Okada M Lin CY Chin K . Nivolumab versus chemotherapy in advanced esophageal squamous cell carcinoma (ESCC): The phase III ATTRACTION-3 study. Ann Oncol (2019) 30:v873–v4. doi: 10.1093/annonc/mdz394.028

[30] Fuchs CS Ozguroglu M Bang YJ Di Bartolomeo M Mandala M Ryu MH . Pembrolizumab (pembro) vs paclitaxel (PTX) for previously treated advanced gastric or gastroesophageal junction (G/GEJ) cancer: Phase 3 KEYNOTE-061 trial. J Clin Oncol (2018) 36(15):4062. doi: 10.1200/JCO.2018.36.15_suppl.4062

[31] Kang YK Boku N Satoh T Ryu MH Chao Y Kato K . Nivolumab in patients with advanced gastric or gastro-oesophageal junction cancer refractory to, or intolerant of, at least two previous chemotherapy regimens (ONO-4538-12, ATTRACTION-2): A randomised, double-blind, placebo-controlled, phase 3 trial. Lancet (2017) 390(10111):2461–71. doi: 10.1016/S0140-6736(17)31827-5 28993052

[32] Kato K Sun JM Shah MA Enzinger PC Adenis A Doi T . Pembrolizumab plus chemotherapy versus chemotherapy as first-line therapy in patients with advanced esophageal cancer: The phase 3 KEYNOTE-590 study. Ann Oncol (2020) 31:S1192–S3. doi: 10.1016/j.annonc.2020.08.2298

[33] Kelly RJ Ajani JA Kuzdzal J Zander T Van Cutsem E Piessen G . Adjuvant nivolumab in resected esophageal or gastroesophageal junction cancer (EC/GEJC) following neoadjuvant chemoradiation therapy (CRT): First results of the CheckMate 577 study. Ann Oncol (2020) 31:S1193–S4. doi: 10.1056/NEJMoa2032125

[34] Moehler MH Cho JY Kim YH Kim JW Di Bartolomeo M Ajani JA . A randomized, open-label, two-arm phase II trial comparing the efficacy of sequential ipilimumab (ipi) versus best supportive care (BSC) following first-line (1L) chemotherapy in patients with unresectable, locally advanced/metastatic (A/M) gastric or gastro-esophageal junction (G/GEJ) cancer. J Clin Oncol (2016) 34:4011. doi: 10.1200/JCO.2016.34.15_suppl.4011

[35] Moehler MH Dvorkin M Ozguroglu M Ryu MH Muntean AS Lonardi S . Results of the JAVELIN gastric 100 phase 3 trial: Avelumab maintenance following first-line (1L) chemotherapy (CTx) vs continuation of CTx for HER2-advanced gastric or gastroesophageal junction cancer (GC/GEJC). J Clin Oncol (2020) 38(4):278. doi: 10.1200/JCO.2020.38.4_suppl.278

[36] Shitara K Özgüroglu M Bang YJ Di Bartolomeo M Mandala M Ryu MH . KEYNOTE-061: Phase 3 study of pembrolizumab vs paclitaxel for previously treated advanced gastric or gastroesophageal junction (G/GEJ) cancer. Ann Oncol (2018) 29:v122. doi: 10.1093/annonc/mdy208.004

[37] Shitara K Özgüroğlu M Bang YJ Di Bartolomeo M Mandalà M Ryu MH . Pembrolizumab versus paclitaxel for previously treated, advanced gastric or gastro-oesophageal junction cancer (KEYNOTE-061): A randomised, open-label, controlled, phase 3 trial. Lancet (2018) 392(10142):123–33. doi: 10.1016/S0140-6736(18)31257-1 29880231

[38] Xu J Li Y Fan Q Shu Y Wu Z Cui T . Sintilimab in patients with advanced esophageal squamous cell carcinoma refractory to previous chemotherapy: A randomized, open-label phase II trial (ORIENT-2). J Clin Oncol (2020) 38(15):4511. doi: 10.1200/JCO.2020.38.15_suppl.4511

[39] Xu RH Luo HY Lu J Bai YX Mao T Wang J . ESCORT-1st: A randomized, double-blind, placebo-controlled, phase 3 trial of camrelizumab plus chemotherapy versus chemotherapy in patients with untreated advanced or metastatic esophageal squamous cell carcinoma (ESCC). J Clin Oncol (2021) 39(15):4000. doi: 10.1200/JCO.2021.39.15_suppl.4000 34875201

[40] Kato K Satoh T Muro K Yoshikawa T Tamura T Hamamoto Y . A subanalysis of Japanese patients in a randomized, double-blind, placebo-controlled, phase 3 trial of nivolumab for patients with advanced gastric or gastro-esophageal junction cancer refractory to, or intolerant of, at least two previous chemotherapy regimens (ONO-4538-12, ATTRACTION-2). Gastric Cancer (2019) 22(2):344–54. doi: 10.1007/s10120-018-0899-6 PMC6394726 30506519

[41] Muro K Kojima T Moriwaki T Kato K Nagashima F Kawakami H . Second-line pembrolizumab versus chemotherapy in Japanese patients with advanced esophageal cancer: subgroup analysis from KEYNOTE-181. Esophagus (2022) 19(1):137–45. doi: 10.1007/s10388-021-00877-3 PMC8739314 34591237

[42] Satoh T Kang YK Chao Y Ryu MH Kato K Cheol Chung H . Exploratory subgroup analysis of patients with prior trastuzumab use in the ATTRACTION-2 trial: A randomized phase III clinical trial investigating the efficacy and safety of nivolumab in patients with advanced gastric/gastroesophageal junction cancer. Gastric Cancer (2020) 23(1):143–53. doi: 10.1007/s10120-019-00970-8 PMC6942596 31087200

[43] Takahashi M Kato K Okada M Chin K Kadowaki S Hamamoto Y . Nivolumab versus chemotherapy in Japanese patients with advanced esophageal squamous cell carcinoma: A subgroup analysis of a multicenter, randomized, open-label, phase 3 trial (ATTRACTION-3). Esophagus (2021) 18(1):90–9. doi: 10.1007/s10388-020-00794-x PMC7794205 33170461

[44] Metges JP Kato K Sun JM Shah MA Enzinger PC Adenis A . First-line pembrolizumab plus chemotherapy versus chemotherapy in advanced esophageal cancer: Longer-term efficacy, safety, and quality-of-life results from the phase 3 KEYNOTE-590 study. J Clin Oncol (2022) 40(4 SUPPL):241. doi: 10.1200/JCO.2022.40.4_suppl.241

[45] Shitara K Özgüroğlu M Bang YJ Di Bartolomeo M Mandalà M Ryu MH . Molecular determinants of clinical outcomes with pembrolizumab versus paclitaxel in a randomized, open-label, phase III trial in patients with gastroesophageal adenocarcinoma. Ann Oncol (2021) 32(9):1127–36. doi: 10.1016/j.annonc.2021.05.803 34082019

[46] Zander T Kelly RJ Ajani JA Kuzdzal J Van Cutsem E Piessen G . Adjuvant nivolumab (NIVO) in resected esophageal or gastroesophageal junction cancer (EC/GEJC) following neoadjuvant chemoradiotherapy (CRT): Expanded efficacy and safety analyses from CheckMate 577. Oncol Res Treat (2021) 44(SUPPL 2):80–1. doi: 10.1159/000518417

[47] Zhao JJ Yap DWT Chan YH Tan BKJ Teo CB Syn NL . Low programmed death-ligand 1-expressing subgroup outcomes of first-line immune checkpoint inhibitors in gastric or esophageal adenocarcinoma. J Clin Oncol (2022) 40(4):392–402. doi: 10.1200/JCO.21.01862 34860570

[48] Cao Y Qin S Luo S Li Z Cheng Y Fan Y . Pembrolizumab versus chemotherapy for patients with esophageal squamous cell carcinoma enrolled in the randomized KEYNOTE-181 trial in Asia. ESMO Open (2022) 7(1):100341. doi: 10.1016/j.esmoop.2021.100341 34973513 PMC8764510

[49] Doki Y Ajani JA Kato K Xu J Wyrwicz L Motoyama S . Nivolumab combination therapy in advanced esophageal squamous-cell carcinoma. N Engl J Med (2022) 386(5):449–62. doi: 10.1056/NEJMoa2111380 35108470

[50] Huang J Xu J Chen Y Zhuang W Zhang Y Chen Z . Camrelizumab versus investigator's choice of chemotherapy as second-line therapy for advanced or metastatic oesophageal squamous cell carcinoma (ESCORT): A multicentre, randomised, open-label, phase 3 study. Lancet Oncol (2020) 21(6):832–42. doi: 10.1016/S1470-2045(20)30110-8 32416073

[51] Kato K Cho BC Takahashi M Okada M Lin CY Chin K . Nivolumab versus chemotherapy in patients with advanced oesophageal squamous cell carcinoma refractory or intolerant to previous chemotherapy (ATTRACTION-3): a multicentre, randomised, open-label, phase 3 trial. Lancet Oncol (2019) 20(11):1506–17. doi: 10.1016/S1470-2045(19)30626-6 31582355

[52] Kelly RJ Ajani JA Kuzdzal J Zander T van Cutsem E Piessen G . Adjuvant nivolumab in resected esophageal or gastroesophageal junction cancer. New Engl J Med (2021) 384(13):1191–203. doi: 10.1056/NEJMoa2032125 33789008

[53] Kojima T Shah MA Muro K Francois E Adenis A Hsu CH . Randomized phase III KEYNOTE-181 study of pembrolizumab versus chemotherapy in advanced esophageal cancer. J Clin Oncol (2020) 38(35):4138–48. doi: 10.1200/JCO.20.01888 33026938

[54] Luo H Lu J Bai Y Mao T Wang J Fan Q . Effect of camrelizumab vs placebo added to chemotherapy on survival and progression-free survival in patients with advanced or metastatic esophageal squamous cell carcinoma: The ESCORT-1st randomized clinical trial. JAMA (2021) 326(10):916–25. doi: 10.1001/jama.2021.12836 PMC8441593 34519801

[55] Park S Sun JM Choi YL Oh D Kim HK Lee T . Adjuvant durvalumab for esophageal squamous cell carcinoma after neoadjuvant chemoradiotherapy: A placebo-controlled, randomized, double-blind, phase II study. ESMO Open (2022) 7(1):100385. doi: 10.1016/j.esmoop.2022.100385 35158205 PMC8850741

[56] Sun JM Shen L Shah MA Enzinger P Adenis A Doi T . Pembrolizumab plus chemotherapy versus chemotherapy alone for first-line treatment of advanced oesophageal cancer (KEYNOTE-590): A randomised, placebo-controlled, phase 3 study. Lancet (2021) 398(10302):759–71. doi: 10.1016/S0140-6736(21)01234-4 34454674

[57] Wang ZX Cui C Yao J Zhang Y Li M Feng J . Toripalimab plus chemotherapy in treatment-naïve, advanced esophageal squamous cell carcinoma (JUPITER-06): A multi-center phase 3 trial. Cancer Cell (2022) 40(3):277–88.e3. doi: 10.1016/j.ccell.2022.02.007 35245446

[58] Xu J Li Y Fan Q Shu Y Yang L Cui T . Clinical and biomarker analyses of sintilimab versus chemotherapy as second-line therapy for advanced or metastatic esophageal squamous cell carcinoma: a randomized, open-label phase 2 study (ORIENT-2). Nat Commun (2022) 13(1):857. doi: 10.1038/s41467-022-28408-3 35165274 PMC8844279

[59] Fusi A Festino L Botti G Masucci G Melero I Lorigan P . PD-L1 expression as a potential predictive biomarker. Lancet Oncol (2015) 16(13):1285–7. doi: 10.1016/S1470-2045(15)00307-1 26433815

[60] Zhang B Liu Y Zhou S Jiang H Zhu K Wang R . Predictive effect of PD-L1 expression for immune checkpoint inhibitor (PD-1/PD-L1 inhibitors) treatment for non-small cell lung cancer: A meta-analysis. Int Immunopharmacol (2020) 80:106214. doi: 10.1016/j.intimp.2020.106214 31982822

[61] Naumann RW Hollebecque A Meyer T Devlin M-J Oaknin A Kerger J . Safety and efficacy of nivolumab monotherapy in recurrent or metastatic cervical, vaginal, or vulvar carcinoma: Results from the phase I/II CheckMate 358 trial. J Clin Oncol (2019) 37(31):2825. doi: 10.1200/JCO.19.00739 31487218 PMC6823884

[62] Wang C H-n W Wang L . Biomarkers for predicting the efficacy of immune checkpoint inhibitors. J Cancer (2022) 13(2):481. doi: 10.7150/jca.65012 35069896 PMC8771507

[63] Paz-Ares L Horn L Borghaei H Spigel DR Steins M Ready N . Phase III, randomized trial (CheckMate 057) of nivolumab (NIVO) versus docetaxel (DOC) in advanced non-squamous cell (non-SQ) non-small cell lung cancer (NSCLC). Am Soc Clin Oncol (2015) 33(18_suppl). doi: 10.1200/jco.2015.33.18_suppl.lba109

[64] Brahmer J Reckamp KL Baas P Crinò L Eberhardt WE Poddubskaya E . Nivolumab versus docetaxel in advanced squamous-cell non–small-cell lung cancer. New Engl J Med (2015) 373(2):123–35. doi: 10.1056/NEJMoa1504627 PMC4681400 26028407

[65] Vilain RE Menzies AM Wilmott JS Kakavand H Madore J Guminski A . Dynamic changes in PD-L1 expression and immune infiltrates early during treatment predict response to PD-1 blockade in MelanomaPD-1 inhibition in melanoma. Clin Cancer Res (2017) 23(17):5024–33. doi: 10.1158/1078-0432.ccr-16-0698 28512174

[66] Shieh KR Huang A Xu Y . Response to immune checkpoint inhibitor treatment in advanced cervical cancer and biomarker study. Front Med (2021) 1184. doi: 10.3389/fmed.2021.669587 PMC8387671 34458284

[67] Tabernero J Van Cutsem E Bang Y-J Fuchs CS Wyrwicz L Lee KW . Pembrolizumab with or without chemotherapy versus chemotherapy for advanced gastric or gastroesophageal junction (G/GEJ) adenocarcinoma: The phase III KEYNOTE-062 study. Am Soc Clin Oncol (2019) 30:iv152-3. doi: 10.1093/annonc/mdz183.001

[68] Wainberg ZA Fuchs CS Tabernero J Shitara K Muro K Van Cutsem E . Efficacy of pembrolizumab monotherapy for advanced Gastric/Gastroesophageal junction cancer with programmed death ligand 1 combined positive score≥ 10Efficacy of pembrolizumab for CPS 10 G/GEJ cancer. Clin Cancer Res (2021) 27(7):1923–31. doi: 10.1158/1078-0432.CCR-20-2980 33446564

[69] Shitara K Ajani JA Moehler M Garrido M Gallardo C Shen L . Nivolumab plus chemotherapy or ipilimumab in gastro-oesophageal cancer. Nature (2022) 603(7903):942–8. doi: 10.1038/s41586-022-04508-4 PMC8967713 35322232

[70] Matsubara Y Toriyama K Kadowaki S Ogata T Nakazawa T Kato K . The impact of combined PD-L1 positive score on clinical response to nivolumab in patients with advanced esophageal squamous cell carcinoma. (2022). doi: 10.21203/rs.3.rs-1468822/v1 36595124

[71] Zou Y Zou X Zheng S Tang H Zhang L Liu P . Efficacy and predictive factors of immune checkpoint inhibitors in metastatic breast cancer: A systematic review and meta-analysis. Ther Adv Med Oncol (2020) 12:1758835920940928. doi: 10.1177/1758835920940928 32874208 PMC7436841

[72] Zhou Y Chen C Zhang X Fu S Xue C Ma Y . Immune-checkpoint inhibitor plus chemotherapy versus conventional chemotherapy for first-line treatment in advanced non-small cell lung carcinoma: A systematic review and meta-analysis. J Immunother Cancer (2018) 6(1):1–11. doi: 10.1186/s40425-018-0477-9 30577837 PMC6303974

[73] Yang JJ Huang C Fan Y Pan H Feng J Jiang L . Camrelizumab in different PD-L1 expression cohorts of pre-treated advanced or metastatic non-small cell lung cancer: A phase II study. Cancer Immunol Immunother (2022) 71(6):1393–402. doi: 10.1007/s00262-021-03091-3 PMC10991208 34668977

[74] Park S Choi YD Kim J Kho BG Park CK Oh IJ . Efficacy of immune checkpoint inhibitors according to PD-L1 tumor proportion scores in non-small cell lung cancer. Thorac Cancer (2020) 11(2):408–14. doi: 10.1111/1759-7714.13284 PMC6996995 31841269

[75] Oh S Kim E Lee H . Comparative impact of PD-1 and PD-L1 inhibitors on advanced esophageal or gastric/gastroesophageal junction cancer treatment: A systematic review and meta-analysis. J Clin Med (2021) 10(16):3612. doi: 10.3390/jcm10163612 34441907 PMC8397221

[76] Mori K Abufaraj M Mostafaei H Quhal F Fajkovic H Remzi M . The predictive value of programmed death ligand 1 in patients with metastatic renal cell carcinoma treated with immune-checkpoint inhibitors: A systematic review and meta-analysis. Eur Urol (2021) 79(6):783–92. doi: 10.1016/j.eururo.2020.10.006 33172722

