# Supplementary material for: Predictive value of PD-L1 expression in response to immune checkpoint inhibitors for esophageal cancer treatment: A systematic review and meta-analysis
Source: Front Oncol. 2022 Dec 15;12:1021859. doi: 10.3389/fonc.2022.1021859 (PMC9798008; doi:10.3389/fonc.2022.1021859)
Supplement: Supplementary file 1 [file DataSheet_1.pdf]

Predictive value of PD-L1 expression in response to immune checkpoint inhibitors for esophageal cancer treatment; A systematic review and meta-analysis

Table S1. Search strategy.

Database (Search date)	Step	Search strategy	No. of results
**PubMed (3.25.2022)**	#1	"Stomach Neoplasms"[MeSh] OR "Esophageal Neoplasms"[MeSH] OR "Esophageal Squamous Cell Carcinoma"[MeSH] OR "Familial primary gastric lymphoma"[Supplementary Concept] OR "Esophageal Squamous Cell Carcinoma"[tiab] OR "Gastric neoplas*"[tiab] OR "gastric tumor*"[tiab] OR "gastric tumour*" OR "Gastric cancer*"[tiab] OR "Cancer of stomach"[tiab] OR "Stomach neoplas*"[tiab] OR "Stomach cancer*"[tiab] OR "stomach tumor*"[tiab] OR "stomach tumour*"[tiab] OR "Cancer of the stomach"[tiab] OR "Gastroesophageal junction adenocarcinoma"[tiab] OR "Gastroesophageal junction tumor*"[tiab] OR "Gastroesophageal junction tumour*"[tiab] OR "Gastroesophageal junction cancer*"[tiab] OR "Gastroesophageal junction neoplasm*"[tiab] OR "Esophagogastri* junction adenocarcinoma"[tiab] OR "Esophagogastri* junction tumor*"[tiab] OR "Esophagogastri* junction tumour*"[tiab] OR "Esophagogastri* junction cancer*"[tiab] OR "Esophagogastri* junction neoplasm*"[tiab] OR "Esophagogastri* cancer*"[tiab] OR "Esophagogastri* neoplasm*"[tiab] OR "Esophagogastri* tumour*"[tiab] OR "Esophagogastri* tumor*"[tiab] OR "Esophagogastri* adenocarcinoma*"[tiab] OR "Esophagogastri* adenoma*"[tiab] OR "Esophag* cancer*"[tiab] OR "oesophag* cancer*"[tiab] OR "Esophag* neoplasm*"[tiab] OR "oesophag* neoplasm*"[tiab] OR "Esophag* tumour*"[tiab] OR "oesophag* tumour*"[tiab] OR "Esophag* tumor*"[tiab] OR "oesophag* tumor*"[tiab] OR "Esophag* adenoma*"[tiab] OR "Esophag* adenocarcinoma*"[tiab] OR "oesophag* adenoma*"[tiab] OR "oesophag* adenocarcinoma*"[tiab] OR "gastric lymphoma*"[tiab] OR "stomach carcinoma"[tiab] OR "gastric carcinoma"[tiab] OR "gastric adenocarcinoma"[tiab] OR "stomach adenocarcinoma"[tiab] OR "stomach lymphoma*"[tiab] OR "adenocarcinoma of the stomach"[tiab] OR "adenocarcinoma of stomach"[tiab] OR "adenoma of the stomach"[tiab] OR "adenoma of stomach"[tiab] OR "adenocarcinoma of the gastroesophageal junction"[tiab] OR "adenocarcinoma of gastroesophageal junction"[tiab] OR "adenocarcinoma of the esophagus"[tiab] OR "adenocarcinoma of esophagus"[tiab] OR "adenoma of the esophagus"[tiab] OR "adenoma of esophagus"[tiab] OR "Gastroesophageal tumor*"[tiab] OR "Gastroesophageal tumour*"[tiab] OR "Gastroesophageal cancer*"[tiab] OR "Gastroesophageal neoplasm*"[tiab] OR "Gastroesophageal adenocarcinoma*"[tiab] OR "Gastroesophageal carcinoma*"[tiab] OR "Gastro-esophageal tumor*"[tiab] OR "Gastro-esophageal tumour*"[tiab] OR "Gastro-esophageal cancer*"[tiab] OR "Gastro-esophageal neoplasm*"[tiab] OR "Gastro-esophageal adenocarcinoma*"[tiab] OR "Gastro-esophageal carcinoma*"[tiab] OR "cancer of stomach"[tiab] OR "neoplasm of stomach"[tiab] OR "tumor of stomach"[tiab] OR "tomour of stomach"[tiab] OR "cancer of esophagus"[tiab] OR "neoplasm of esophagus"[tiab] OR "tumor of esophagus"[tiab] OR "tomour of esophagus"[tiab] OR "gastroesophageal junction"[tiab]	185,452
	#2	"Immune Checkpoint Inhibitors"[MeSH] OR "Immune Checkpoint Inhibitor*"[tiab] OR "Immune Checkpoint Blocker*"[tiab] OR "Immune Checkpoint Blockade*"[tiab] OR "Immune Checkpoint Inhibition*"[tiab] OR "PD-L1 Inhibitor*"[tiab] OR "PD L1 Inhibitor*"[tiab] OR "PDL1 Inhibitor*"[tiab] OR "PD-L1 blocker*"[tiab] OR "PD L1 blocker*"[tiab] OR "PDL1 blocker*"[tiab] OR "Programmed Death-Ligand 1 Inhibitor*"[tiab] OR "Programmed Death Ligand 1 Inhibitor*"[tiab] OR "Programmed Death-Ligand 1 blocker*"[tiab] OR "Programmed Death Ligand 1 blocker*"[tiab] OR "anti Programmed Death-Ligand 1"[tiab] OR "anti-Programmed Death-Ligand 1"[tiab] OR "anti Programmed Death Ligand 1"[tiab] OR "anti-Programmed Death Ligand 1"[tiab] OR "CTLA-4 Inhibitor*"[tiab] OR "CTLA 4 Inhibitor*"[tiab] OR "CTLA4 Inhibitor*"[tiab] OR "CTLA-4 blocker*"[tiab] OR "CTLA 4 blocker*"[tiab] OR "CTLA4 blocker*"[tiab] OR "Cytotoxic T-Lymphocyte Associated Protein 4 Inhibitor*"[tiab] OR "Cytotoxic T Lymphocyte-Associated Protein 4 Inhibitor*"[tiab] OR "Cytotoxic T-Lymphocyte Associated Protein 4 blocker*"[tiab] OR "Cytotoxic T Lymphocyte-Associated Protein 4 blocker*"[tiab] OR "Cytotoxic T-Lymphocyte Associated antigen 4 Inhibitor*"[tiab] OR "Cytotoxic T Lymphocyte-Associated antigen 4 Inhibitor*"[tiab] OR "Cytotoxic T-Lymphocyte Associated antigen 4 blocker*"[tiab] OR "Cytotoxic T Lymphocyte-Associated antigen 4 blocker*"[tiab] OR "Cytotoxic T-Lymphocyte-Associated Protein 4 Inhibitor*"[tiab] OR "Cytotoxic T Lymphocyte Associated Protein 4 Inhibitor*"[tiab] OR "Cytotoxic T-Lymphocyte-Associated Protein 4 blocker*"[tiab] OR "Cytotoxic T Lymphocyte Associated Protein 4 blocker*"[tiab] OR "Cytotoxic T-Lymphocyte-Associated antigen 4 Inhibitor*"[tiab] OR "Cytotoxic T Lymphocyte Associated antigen 4 Inhibitor*"[tiab] OR "Cytotoxic T-Lymphocyte-Associated antigen 4 blocker*"[tiab] OR "Cytotoxic T Lymphocyte Associated antigen 4 blocker*"[tiab] OR "anti-cytotoxic T lymphocyte-associated antigen 4"[tiab] OR "anti cytotoxic T lymphocyte-associated antigen 4"[tiab] OR "anti-cytotoxic T lymphocyte-associated protein 4"[tiab] OR "anti cytotoxic T lymphocyte-associated protein 4"[tiab] OR "anti-cytotoxic T-lymphocyte-associated antigen 4"[tiab] OR "anti-cytotoxic T-lymphocyte-associated antigen 4"[tiab] OR "PD-1 Inhibitor*"[tiab] OR "PD 1 Inhibitor*"[tiab] OR "PD1 Inhibitor*"[tiab] OR "PD 1 blocker*"[tiab] OR "PD1 blocker*"[tiab] OR "PD-1 blocker*"[tiab] OR "Programmed Cell Death Protein 1 Inhibitor*"[tiab] OR "Programmed Cell Death Protein 1 blocker*"[tiab] OR "anti Programmed Cell Death Protein 1"[tiab] OR "anti-Programmed Cell Death Protein 1"[tiab] OR "anti-PD1"[tiab] OR "anti PD1"[tiab] OR "anti-PD-1"[tiab] OR "anti PD-1"[tiab] OR "anti-PD-L1"[tiab] OR "anti PDL1"[tiab] OR "anti PD-L1"[tiab] OR "anti-PDL1"[tiab] OR "anti-PD L1"[tiab] OR	38,035

		“anti PD L1”[tiab] OR “anti-CTLA4”[tiab] OR “anti-CTLA 4”[tiab] OR “anti-CTLA-4”[tiab] OR “anti CTLA4”[tiab] OR “anti CTLA 4”[tiab] OR “anti CTLA-4”[tiab] OR “PD-1-PD-L1 Blockade*”[tiab] OR “PD 1 PD L1 Blockade*”[tiab] OR “Pembrolizumab”[tiab] OR “MK-3475”[tiab] OR “lambrolizumab”[tiab] OR “Keytruda”[tiab] OR “SCH-900475”[tiab] OR “Nivolumab”[tiab] OR “Opdivo”[tiab] OR “ONO-4538”[tiab] OR “ONO 4538”[tiab] OR “ONO4538”[tiab] OR “MDX-1106”[tiab] OR “MDX 1106”[tiab] OR “MDX1106”[tiab] OR “BMS-936558”[tiab] OR “BMS 936558”[tiab] OR “BMS936558”[tiab] OR “Ipilimumab”[tiab] OR “Yervoy”[tiab] OR “MDX 010”[tiab] OR “MDX010”[tiab] OR “MDX-010”[tiab] OR “MDX-CTLA-4”[tiab] OR “MDX CTLA 4”[tiab] OR “Durvalumab”[tiab] OR “MEDI4736”[tiab] OR “MEDI-4736”[tiab] OR “Imfinzi”[tiab] OR “Dostarlimab”[tiab] OR “GSK4057190”[tiab] OR “TSR-042”[tiab] OR “Cemiplimab”[tiab] OR ”REGN2810”[tiab] OR “Avelumab”[tiab] OR “MSB-0010682”[tiab] OR “MSB0010682”[tiab] OR ”bavencio”[tiab] OR ”MSB0010718C”[tiab] OR ”MSB-0010718C”[tiab] OR “Atezolizumab”[tiab] OR “MPDL3280A”[tiab] OR ”MPDL-3280A”[tiab] OR “Tecentriq”[tiab] OR “RG7446”[tiab] OR “RG-7446”[tiab] OR “pidilizumab”[tiab] OR “CT-011”[tiab] OR “CT 011”[tiab] OR “tremelimumab”[tiab] OR “ticilimumab”[tiab] OR “CP 675”[tiab] OR “CP675 cpd”[tiab] OR “CP-675”[tiab] OR “CP-675,206”[tiab] OR “CP-675206”[tiab] OR “CP675206”[tiab] OR “CP 675206”[tiab] OR "dostarlimab"[Supplementary Concept] OR "cemiplimab"[Supplementary Concept] OR "Ipilimumab"[Mesh] OR "tremelimumab"[Supplementary Concept] OR "pembrolizumab"[Supplementary Concept] OR "atezolizumab"[Supplementary Concept] OR "Nivolumab"[Mesh] OR "durvalumab"[Supplementary Concept] OR "avelumab" [Supplementary Concept] OR "pidilizumab" [Supplementary Concept] OR “sintilimab”[tiab] OR “SHR-1210”[taib] OR “camrelizumab”[tiab] OR “toripalimab”[tiab] OR “HX008”[tiab]	
	#3	“RCT”[tiab] OR “trial*”[tiab] OR “intervention*”[tiab] OR “placebo”[tiab] OR “randomised trial*”[tiab] OR “randomized trial*”[tiab] OR “controlled trial*”[tiab] OR "Random Allocation"[Mesh] OR "Single-Blind Method"[Mesh] OR "Double-Blind Method"[Mesh] OR "Cross-Over Studies"[Mesh] OR "Clinical Trial"[Publication Type] OR "Clinical Trials as Topic"[Mesh] OR "Controlled Clinical Trial"[Publication Type] OR "Clinical Trial, Phase I"[Publication Type] OR "Clinical Trial, Phase II"[Publication Type] OR "Clinical Trial, Phase III"[Publication Type] OR "Clinical Trial, Phase IV"[Publication Type] OR "Non-Randomized Controlled Trials as Topic"[Mesh]	2,928,066
	#4	“Review”[Publication Type] OR “Review Literature as Topic”[MeSH] OR “Systematic Review”[Publication Type] OR “Systematic Reviews as Topic”[MeSH] OR “Meta-Analysis”[Publication Type] OR “Meta-Analysis as Topic”[MeSH] OR “Network Meta-Analysis”[MeSH]	3,130,061
	#5	#1 AND #2 AND #3 NOT #4	282
**Scopus (3.25.2022)**	#1	TITLE-ABS-KEY(“Esophageal Squamous Cell Carcinoma” OR “Familial primary gastric lymphoma” OR “Esophageal Squamous Cell Carcinoma” OR “Gastric neoplas*” OR “gastric tumor*” OR “gastric tumour*” OR “Gastric cancer*” OR “Cancer of stomach” OR “Stomach neoplas*” OR “Stomach cancer*” OR “stomach tumor*” OR “stomach tumour*” OR “Cancer of the stomach” OR “Gastroesophageal junction adenocarcinoma” OR “Gastroesophageal junction tumor*” OR “Gastroesophageal junction tumour*” OR “Gastroesophageal junction cancer*” OR “Gastroesophageal junction neoplasm*” OR “Esophagogastri* junction adenocarcinoma” OR “Esophagogastri* junction tumor*” OR “Esophagogastri* junction tumour*” OR “Esophagogastri* junction cancer*” OR “Esophagogastri* junction neoplasm*” OR “Esophagogastri* cancer*” OR “Esophagogastri* neoplasm*” OR “Esophagogastri* tumour*” OR “Esophagogastri* tumor*” OR “Esophagogastri* adenocarcinoma*” OR “Esophagogastri* adenoma*” OR “Esophag* cancer*” OR “oesophag* cancer*” OR “Esophag* neoplasm*” OR “oesophag* neoplasm*” OR “Esophag* tumour*” OR “oesophag* tumour*” OR “Esophag* tumor*” OR “oesophag* tumor*” OR “Esophag* adenoma*” OR “Esophag* adenocarcinoma*” OR “oesophag* adenoma*” OR “oesophag* adenocarcinoma*” OR “gastric lymphoma*” OR “stomach carcinoma” OR “gastric carcinoma” OR “gastric adenocarcinoma” OR “stomach adenocarcinoma” OR “stomach lymphoma*” OR “adenocarcinoma of the stomach” OR “adenocarcinoma of stomach” OR “adenoma of the stomach” OR “adenoma of stomach” OR “adenocarcinoma of the gastroesophageal junction” OR “adenocarcinoma of gastroesophageal junction” OR “adenocarcinoma of the esophagus” OR “adenocarcinoma of esophagus” OR “adenoma of the esophagus” OR “adenoma of esophagus” OR “Gastroesophageal tumor*” OR “Gastroesophageal tumour*” OR “Gastroesophageal cancer*” OR “Gastroesophageal neoplasm*” OR “Gastroesophageal adenocarcinoma*” OR “Gastroesophageal carcinoma*” OR “Gastro-esophageal tumor*” OR “Gastro-esophageal tumour*” OR “Gastro-esophageal cancer*” OR “Gastro-esophageal neoplasm*” OR “Gastro-esophageal adenocarcinoma*” OR “Gastro-esophageal carcinoma*” OR “cancer of stomach” OR “neoplasm of stomach” OR “tumor of stomach” OR “tomour of stomach” OR “cancer of esophagus” OR “neoplasm of esophagus” OR “tumor of esophagus” OR “tomour of esophagus” OR “gastroesophageal junction”)	253,744
	#2	TITLE-ABS-KEY(“Immune Checkpoint Inhibitor*” OR “Immune Checkpoint Blocker*” OR “Immune Checkpoint Blockade*” OR “Immune Checkpoint Inhibition*” OR “PD-L1 Inhibitor*” OR “PD L1 Inhibitor*” OR “PDL1 Inhibitor*” OR “PD-L1 blocker*” OR “PD L1 blocker*” OR “PDL1 blocker*” OR	53,854

		"Programmed Death-Ligand 1 Inhibitor*" OR "Programmed Death Ligand 1 Inhibitor*" OR "Programmed Death-Ligand 1 blocker*" OR "Programmed Death Ligand 1 blocker*" OR "anti Programmed Death-Ligand 1" OR "anti-Programmed Death-Ligand 1" OR "anti Programmed Death Ligand 1" OR "anti-Programmed Death Ligand 1" OR "CTLA-4 Inhibitor*" OR "CTLA 4 Inhibitor*" OR "CTLA4 Inhibitor*" OR "CTLA-4 blocker*" OR "CTLA 4 blocker*" OR "CTLA4 blocker*" OR "Cytotoxic T-Lymphocyte Associated Protein 4 Inhibitor*" OR "Cytotoxic T Lymphocyte-Associated Protein 4 Inhibitor*" OR "Cytotoxic T-Lymphocyte Associated Protein 4 blocker*" OR "Cytotoxic T Lymphocyte-Associated Protein 4 blocker*" OR "Cytotoxic T-Lymphocyte Associated antigen 4 Inhibitor*" OR "Cytotoxic T Lymphocyte-Associated antigen 4 Inhibitor*" OR "Cytotoxic T-Lymphocyte Associated antigen 4 blocker*" OR "Cytotoxic T Lymphocyte-Associated antigen 4 blocker*" OR "Cytotoxic T-Lymphocyte-Associated Protein 4 Inhibitor*" OR "Cytotoxic T Lymphocyte Associated Protein 4 Inhibitor*" OR "Cytotoxic T-Lymphocyte-Associated Protein 4 blocker*" OR "Cytotoxic T Lymphocyte Associated Protein 4 blocker*" OR "Cytotoxic T-Lymphocyte-Associated antigen 4 Inhibitor*" OR "Cytotoxic T Lymphocyte Associated antigen 4 Inhibitor*" OR "Cytotoxic T-Lymphocyte-Associated antigen 4 blocker*" OR "Cytotoxic T Lymphocyte Associated antigen 4 blocker*" OR "anti-cytotoxic T lymphocyte-associated antigen 4" OR "anti cytotoxic T lymphocyte-associated antigen 4" OR "anti-cytotoxic T lymphocyte-associated protein 4" OR "anti cytotoxic T lymphocyte-associated protein 4" OR "anti-cytotoxic T-lymphocyte-associated antigen 4" OR "anti-cytotoxic T-lymphocyte-associated antigen 4" OR "PD-1 Inhibitor*" OR "PD 1 Inhibitor*" OR "PD1 Inhibitor*" OR "PD 1 blocker*" OR "PD1 blocker*" OR "PD-1 blocker*" OR "Programmed Cell Death Protein 1 Inhibitor*" OR "Programmed Cell Death Protein 1 blocker*" OR "anti Programmed Cell Death Protein 1" OR "anti-Programmed Cell Death Protein 1" OR "anti-PD1" OR "anti PD1" OR "anti-PD-1" OR "anti PD-1" OR "anti-PD-L1" OR "anti PDL1" OR "anti PD-L1" OR "anti-PDL1" OR "anti-PD L1" OR "anti PD L1" OR "anti-CTLA4" OR "anti-CTLA 4" OR "anti-CTLA-4" OR "anti CTLA4" OR "anti CTLA 4" OR "anti CTLA-4" OR "PD-1-PD-L1 Blockade*" OR "PD 1 PD L1 Blockade*" OR "Pembrolizumab" OR "MK-3475" OR "lambrolizumab" OR "Keytruda" OR "SCH-900475" OR "Nivolumab" OR "Opdivo" OR "ONO-4538" OR "ONO 4538" OR "ONO4538" OR "MDX-1106" OR "MDX 1106" OR "MDX1106" OR "BMS-936558" OR "BMS 936558" OR "BMS936558" OR "Ipilimumab" OR "Yervoy" OR "MDX 010" OR "MDX010" OR "MDX-010" OR "MDX-CTLA-4" OR "MDX CTLA 4" OR "Durvalumab" OR "MEDI4736" OR "MEDI-4736" OR "Imfinzi" OR "Dostarlimab" OR "GSK4057190" OR "TSR-042" OR "Cemiplimab" OR "REGN2810" OR "Avelumab" OR "MSB-0010682" OR "MSB0010682" OR "bavencio" OR "MSB0010718C" OR "MSB-0010718C" OR "Atezolizumab" OR "MPDL3280A" OR "MPDL-3280A" OR "Tecentriq" OR "RG7446" OR "RG-7446" OR "pidilizumab" OR "CT-011" OR "CT 011" OR "tremelimumab" OR "ticilimumab" OR "CP 675" OR "CP675 cpd" OR "CP-675" OR "CP-675,206" OR "CP-675206" OR "CP675206" OR "CP 675206" OR "sintilimab" OR "SHR-1210" OR "camrelizumab" OR "toripalimab" OR "HX008")	
	#3	TITLE-ABS-KEY("RCT" OR "trial*" OR "intervention*" OR "placebo" OR "randomised trial*" OR "randomized trial*" OR "controlled trial*" OR "Random Allocation" OR "Single-Blind" OR "Double-Blind" OR "Cross-Over" OR "Clinical Trial*" OR "Controlled Clinical Trial" OR "Non-Randomized Controlled Trial*")	4,254,341
	#4	TITLE-ABS-KEY("Review" OR "Review of Literature" OR "Systematic Review" OR "Meta-Analysis" OR "Meta Analysis" OR "Network Meta-Analysis" OR "Network Meta Analysis")	5,554,924
	#5	#1 AND #2 AND #3 NOT #4	385
**WOS (3.25.2022)**	#1	TS=("Esophageal Squamous Cell Carcinoma" OR "Familial primary gastric lymphoma" OR "Esophageal Squamous Cell Carcinoma" OR "Gastric neoplas*" OR "gastric tumor*" OR "gastric tumour*" OR "Gastric cancer*" OR "Cancer of stomach" OR "Stomach neoplas*" OR "Stomach cancer*" OR "stomach tumor*" OR "stomach tumour*" OR "Cancer of the stomach" OR "Gastroesophageal junction adenocarcinoma" OR "Gastroesophageal junction tumor*" OR "Gastroesophageal junction tumour*" OR "Gastroesophageal junction cancer*" OR "Gastroesophageal junction neoplasm*" OR "Esophagogastri* junction adenocarcinoma" OR "Esophagogastri* junction tumor*" OR "Esophagogastri* junction tumour*" OR "Esophagogastri* junction cancer*" OR "Esophagogastri* junction neoplasm*" OR "Esophagogastri* cancer*" OR "Esophagogastri* neoplasm*" OR "Esophagogastri* tumour*" OR "Esophagogastri* tumor*" OR "Esophagogastri* adenocarcinoma*" OR "Esophagogastri* adenoma*" OR "Esophag* cancer*" OR "oesophag* cancer*" OR "Esophag* neoplasm*" OR "oesophag* neoplasm*" OR "Esophag* tumour*" OR "oesophag* tumour*" OR "Esophag* tumor*" OR "oesophag* tumor*" OR "Esophag* adenoma*" OR "Esophag* adenocarcinoma*" OR "oesophag* adenoma*" OR "oesophag* adenocarcinoma*" OR "gastric lymphoma*" OR "stomach carcinoma" OR "gastric carcinoma" OR "gastric adenocarcinoma" OR "stomach adenocarcinoma" OR "stomach lymphoma*" OR "adenocarcinoma of the stomach" OR "adenocarcinoma of stomach" OR "adenoma of the stomach" OR "adenoma of stomach" OR "adenocarcinoma of the gastroesophageal junction" OR "adenocarcinoma of gastroesophageal junction" OR "adenocarcinoma of the esophagus" OR "adenocarcinoma of esophagus" OR "adenoma of the esophagus" OR "adenoma of esophagus" OR "Gastroesophageal tumor*" OR "Gastroesophageal tumour*" OR "Gastroesophageal cancer*" OR "Gastroesophageal	159,250

		neoplasm*" OR "Gastroesophageal adenocarcinoma*" OR "Gastroesophageal carcinoma*" OR "Gastro-esophageal tumor*" OR "Gastro-esophageal tumour*" OR "Gastro-esophageal cancer*" OR "Gastro-esophageal neoplasm*" OR "Gastro-esophageal adenocarcinoma*" OR "Gastro-esophageal carcinoma*" OR "cancer of stomach" OR "neoplasm of stomach" OR "tumor of stomach" OR "tomour of stomach" OR "cancer of esophagus" OR "neoplasm of esophagus" OR "tumor of esophagus" OR "tomour of esophagus" OR "gastroesophageal junction")	
	#2	TS=("Immune Checkpoint Inhibitor*" OR "Immune Checkpoint Blocker*" OR "Immune Checkpoint Blockade*" OR "Immune Checkpoint Inhibition*" OR "PD-L1 Inhibitor*" OR "PD L1 Inhibitor*" OR "PDL1 Inhibitor*" OR "PD-L1 blocker*" OR "PD L1 blocker*" OR "PDL1 blocker*" OR "Programmed Death-Ligand 1 Inhibitor*" OR "Programmed Death Ligand 1 Inhibitor*" OR "Programmed Death-Ligand 1 blocker*" OR "Programmed Death Ligand 1 blocker*" OR "anti Programmed Death-Ligand 1" OR "anti-Programmed Death-Ligand 1" OR "anti Programmed Death Ligand 1" OR "anti-Programmed Death Ligand 1" OR "CTLA-4 Inhibitor*" OR "CTLA 4 Inhibitor*" OR "CTLA4 Inhibitor*" OR "CTLA-4 blocker*" OR "CTLA 4 blocker*" OR "CTLA4 blocker*" OR "Cytotoxic T-Lymphocyte Associated Protein 4 Inhibitor*" OR "Cytotoxic T Lymphocyte-Associated Protein 4 Inhibitor*" OR "Cytotoxic T-Lymphocyte Associated Protein 4 blocker*" OR "Cytotoxic T Lymphocyte-Associated Protein 4 blocker*" OR "Cytotoxic T-Lymphocyte Associated antigen 4 Inhibitor*" OR "Cytotoxic T Lymphocyte-Associated antigen 4 Inhibitor*" OR "Cytotoxic T-Lymphocyte Associated antigen 4 blocker*" OR "Cytotoxic T Lymphocyte-Associated antigen 4 blocker*" OR "Cytotoxic T-Lymphocyte-Associated Protein 4 Inhibitor*" OR "Cytotoxic T Lymphocyte Associated Protein 4 Inhibitor*" OR "Cytotoxic T-Lymphocyte-Associated Protein 4 blocker*" OR "Cytotoxic T Lymphocyte Associated Protein 4 blocker*" OR "Cytotoxic T-Lymphocyte-Associated antigen 4 Inhibitor*" OR "Cytotoxic T Lymphocyte Associated antigen 4 Inhibitor*" OR "Cytotoxic T-Lymphocyte-Associated antigen 4 blocker*" OR "Cytotoxic T Lymphocyte Associated antigen 4 blocker*" OR "anti-cytotoxic T lymphocyte-associated antigen 4" OR "anti cytotoxic T lymphocyte-associated antigen 4" OR "anti-cytotoxic T lymphocyte-associated protein 4" OR "anti cytotoxic T lymphocyte-associated protein 4" OR "anti-cytotoxic T-lymphocyte-associated antigen 4" OR "anti-cytotoxic T-lymphocyte-associated antigen 4" OR "PD-1 Inhibitor*" OR "PD 1 Inhibitor*" OR "PD1 Inhibitor*" OR "PD 1 blocker*" OR "PD1 blocker*" OR "PD-1 blocker*" OR "Programmed Cell Death Protein 1 Inhibitor*" OR "Programmed Cell Death Protein 1 blocker*" OR "anti Programmed Cell Death Protein 1" OR "anti-Programmed Cell Death Protein 1" OR "anti-PD1" OR "anti PD1" OR "anti-PD-1" OR "anti PD-1" OR "anti-PD-L1" OR "anti PDL1" OR "anti PD-L1" OR "anti-PDL1" OR "anti-PD L1" OR "anti PD L1" OR "anti-CTLA4" OR "anti-CTLA 4" OR "anti-CTLA-4" OR "anti CTLA4" OR "anti CTLA 4" OR "anti CTLA-4" OR "PD-1-PD-L1 Blockade*" OR "PD 1 PD L1 Blockade*" OR "Pembrolizumab" OR "MK-3475" OR "lambrolizumab" OR "Keytruda" OR "SCH-900475" OR "Nivolumab" OR "Opdivo" OR "ONO-4538" OR "ONO 4538" OR "ONO4538" OR "MDX-1106" OR "MDX 1106" OR "MDX1106" OR "BMS-936558" OR "BMS 936558" OR "BMS936558" OR "Ipilimumab" OR "Yervoy" OR "MDX 010" OR "MDX010" OR "MDX-010" OR "MDX-CTLA-4" OR "MDX CTLA 4" OR "Durvalumab" OR "MEDI4736" OR "MEDI-4736" OR "Imfinzi" OR "Dostarlimab" OR "GSK4057190" OR "TSR-042" OR "Cemiplimab" OR "REGN2810" OR "Avelumab" OR "MSB-0010682" OR "MSB0010682" OR "bavencio" OR "MSB0010718C" OR "MSB-0010718C" OR "Atezolizumab" OR "MPDL3280A" OR "MPDL-3280A" OR "Tecentriq" OR "RG7446" OR "RG-7446" OR "pidilizumab" OR "CT-011" OR "CT 011" OR "tremelimumab" OR "ticilimumab" OR "CP 675" OR "CP675 cpd" OR "CP-675" OR "CP-675,206" OR "CP-675206" OR "CP675206" OR "CP 675206" OR "sintilimab" OR "SHR-1210" OR "camrelizumab" OR "toripalimab" OR "HX008")	54,795
	#3	TS=("RCT" OR "trial*" OR "intervention*" OR "placebo" OR "randomised trial*" OR "randomized trial*" OR "controlled trial*" OR "Random Allocation" OR "Single-Blind" OR "Double-Blind" OR "Cross-Over" OR "Clinical Trial*" OR "Controlled Clinical Trial" OR "Non-Randomized Controlled Trial*")	2,969,275
	#4	TS=("Review" OR "Review of Literature" OR "Systematic Review" OR "Meta-Analysis" OR "Meta Analysis" OR "Network Meta-Analysis" OR "Network Meta Analysis")	2,325,589
	#5	#1 AND #2 AND #3 NOT #4	488
**Embase (3.25.2022)**	#1	("Esophageal Squamous Cell Carcinoma" OR "Familial primary gastric lymphoma" OR "Esophageal Squamous Cell Carcinoma" OR "Gastric neoplas*" OR "gastric tumor*" OR "gastric tumour*" OR "Gastric cancer*" OR "Cancer of stomach" OR "Stomach neoplas*" OR "Stomach cancer*" OR "stomach tumor*" OR "stomach tumour*" OR "Cancer of the stomach" OR "Gastroesophageal junction adenocarcinoma" OR "Gastroesophageal junction tumor*" OR "Gastroesophageal junction tumour*" OR "Gastroesophageal junction cancer*" OR "Gastroesophageal junction neoplasm*" OR "Esophagogastri* junction adenocarcinoma" OR "Esophagogastri* junction tumor*" OR "Esophagogastri* junction tumour*" OR "Esophagogastri* junction cancer*" OR "Esophagogastri* junction neoplasm*" OR "Esophagogastri* cancer*" OR "Esophagogastri* neoplasm*" OR "Esophagogastri* tumour*" OR "Esophagogastri* tumor*" OR "Esophagogastri* adenocarcinoma*" OR "Esophagogastri* adenoma*" OR "Esophag* cancer*" OR "oesophag* cancer*" OR "Esophag* neoplasm*" OR	195,759

	"oesophag* neoplasm*" OR "Esophag* tumour*" OR "oesophag* tumour*" OR "Esophag* tumor*" OR "oesophag* tumor*" OR "Esophag* adenoma*" OR "Esophag* adenocarcinoma*" OR "oesophag* adenoma*" OR "oesophag* adenocarcinoma*" OR "gastric lymphoma*" OR "stomach carcinoma" OR "gastric carcinoma" OR "gastric adenocarcinoma" OR "stomach adenocarcinoma" OR "stomach lymphoma*" OR "adenocarcinoma of the stomach" OR "adenocarcinoma of stomach" OR "adenoma of the stomach" OR "adenoma of stomach" OR "adenocarcinoma of the gastroesophageal junction" OR "adenocarcinoma of gastroesophageal junction" OR "adenocarcinoma of the esophagus" OR "adenocarcinoma of esophagus" OR "adenoma of the esophagus" OR "adenoma of esophagus" OR "Gastroesophageal tumor*" OR "Gastroesophageal tumour*" OR "Gastroesophageal cancer*" OR "Gastroesophageal neoplasm*" OR "Gastroesophageal adenocarcinoma*" OR "Gastroesophageal carcinoma*" OR "Gastro-esophageal tumor*" OR "Gastro-esophageal tumour*" OR "Gastro-esophageal cancer*" OR "Gastro-esophageal neoplasm*" OR "Gastro-esophageal adenocarcinoma*" OR "Gastro-esophageal carcinoma*" OR "cancer of stomach" OR "neoplasm of stomach" OR "tumor of stomach" OR "tomour of stomach" OR "cancer of esophagus" OR "neoplasm of esophagus" OR "tumor of esophagus" OR "tomour of esophagus" OR "gastroesophageal junction"):ab,ti	
#2	("Immune Checkpoint Inhibitor*" OR "Immune Checkpoint Blocker*" OR "Immune Checkpoint Blockade*" OR "Immune Checkpoint Inhibition*" OR "PD-L1 Inhibitor*" OR "PD L1 Inhibitor*" OR "PDL1 Inhibitor*" OR "PD-L1 blocker*" OR "PD L1 blocker*" OR "PDL1 blocker*" OR "Programmed Death-Ligand 1 Inhibitor*" OR "Programmed Death Ligand 1 Inhibitor*" OR "Programmed Death-Ligand 1 blocker*" OR "Programmed Death Ligand 1 blocker*" OR "anti Programmed Death-Ligand 1" OR "anti-Programmed Death-Ligand 1" OR "anti Programmed Death Ligand 1" OR "anti-Programmed Death Ligand 1" OR "CTLA-4 Inhibitor*" OR "CTLA 4 Inhibitor*" OR "CTLA4 Inhibitor*" OR "CTLA-4 blocker*" OR "CTLA 4 blocker*" OR "CTLA4 blocker*" OR "Cytotoxic T-Lymphocyte Associated Protein 4 Inhibitor*" OR "Cytotoxic T Lymphocyte-Associated Protein 4 Inhibitor*" OR "Cytotoxic T-Lymphocyte Associated Protein 4 blocker*" OR "Cytotoxic T Lymphocyte-Associated Protein 4 blocker*" OR "Cytotoxic T-Lymphocyte Associated antigen 4 Inhibitor*" OR "Cytotoxic T Lymphocyte-Associated antigen 4 Inhibitor*" OR "Cytotoxic T-Lymphocyte Associated antigen 4 blocker*" OR "Cytotoxic T Lymphocyte-Associated antigen 4 blocker*" OR "Cytotoxic T-Lymphocyte-Associated Protein 4 Inhibitor*" OR "Cytotoxic T Lymphocyte Associated Protein 4 Inhibitor*" OR "Cytotoxic T-Lymphocyte-Associated Protein 4 blocker*" OR "Cytotoxic T Lymphocyte Associated Protein 4 blocker*" OR "Cytotoxic T-Lymphocyte-Associated antigen 4 Inhibitor*" OR "Cytotoxic T Lymphocyte Associated antigen 4 Inhibitor*" OR "Cytotoxic T-Lymphocyte-Associated antigen 4 blocker*" OR "Cytotoxic T Lymphocyte Associated antigen 4 blocker*" OR "anti-cytotoxic T lymphocyte-associated antigen 4" OR "anti cytotoxic T lymphocyte-associated antigen 4" OR "anti-cytotoxic T lymphocyte-associated protein 4" OR "anti cytotoxic T lymphocyte-associated protein 4" OR "anti-cytotoxic T-lymphocyte-associated antigen 4" OR "anti-cytotoxic T-lymphocyte-associated antigen 4" OR "PD-1 Inhibitor*" OR "PD 1 Inhibitor*" OR "PD1 Inhibitor*" OR "PD 1 blocker*" OR "PD1 blocker*" OR "PD-1 blocker*" OR "Programmed Cell Death Protein 1 Inhibitor*" OR "Programmed Cell Death Protein 1 blocker*" OR "anti Programmed Cell Death Protein 1" OR "anti-Programmed Cell Death Protein 1" OR "anti-PD1" OR "anti PD1" OR "anti-PD-1" OR "anti PD-1" OR "anti-PD-L1" OR "anti PDL1" OR "anti PD-L1" OR "anti-PDL1" OR "anti-PD L1" OR "anti PD L1" OR "anti-CTLA4" OR "anti-CTLA 4" OR "anti-CTLA-4" OR "anti CTLA4" OR "anti CTLA 4" OR "anti CTLA-4" OR "PD-1-PD-L1 Blockade*" OR "PD 1 PD L1 Blockade*" OR "Pembrolizumab" OR "MK-3475" OR "lambrolizumab" OR "Keytruda" OR "SCH-900475" OR "Nivolumab" OR "Opdivo" OR "ONO-4538" OR "ONO 4538" OR "ONO4538" OR "MDX-1106" OR "MDX 1106" OR "MDX1106" OR "BMS-936558" OR "BMS 936558" OR "BMS936558" OR "Ipilimumab" OR "Yervoy" OR "MDX 010" OR "MDX010" OR "MDX-010" OR "MDX-CTLA-4" OR "MDX CTLA 4" OR "Durvalumab" OR "MEDI4736" OR "MEDI-4736" OR "Imfinzi" OR "Dostarlimab" OR "GSK4057190" OR "TSR-042" OR "Cemiplimab" OR "REGN2810" OR "Avelumab" OR "MSB-0010682" OR "MSB0010682" OR "bavencio" OR "MSB0010718C" OR "MSB-0010718C" OR "Atezolizumab" OR "MPDL3280A" OR "MPDL-3280A" OR "Tecentriq" OR "RG7446" OR "RG-7446" OR "pidilizumab" OR "CT-011" OR "CT 011" OR "tremelimumab" OR "ticilimumab" OR "CP 675" OR "CP675 cpd" OR "CP-675" OR "CP-675,206" OR "CP-675206" OR "CP675206" OR "CP 675206" OR "sintilimab" OR "SHR-1210" OR "camrelizumab" OR "toripalimab" OR "HX008"):ab,ti	69,401
#3	("RCT" OR "trial*" OR "intervention*" OR "placebo" OR "randomised trial*" OR "randomized trial*" OR "controlled trial*" OR "Random Allocation" OR "Single-Blind" OR "Double-Blind" OR "Cross-Over" OR "Clinical Trial*" OR "Controlled Clinical Trial" OR "Non-Randomized Controlled Trial*"):ab,ti	3,273,150
#4	("Review" OR "Review of Literature" OR "Systematic Review" OR "Meta-Analysis" OR "Meta Analysis" OR "Network Meta-Analysis" OR "Network Meta Analysis"):ab,ti	2,540,599
#5	#1 AND #2 AND #3 NOT #4	807

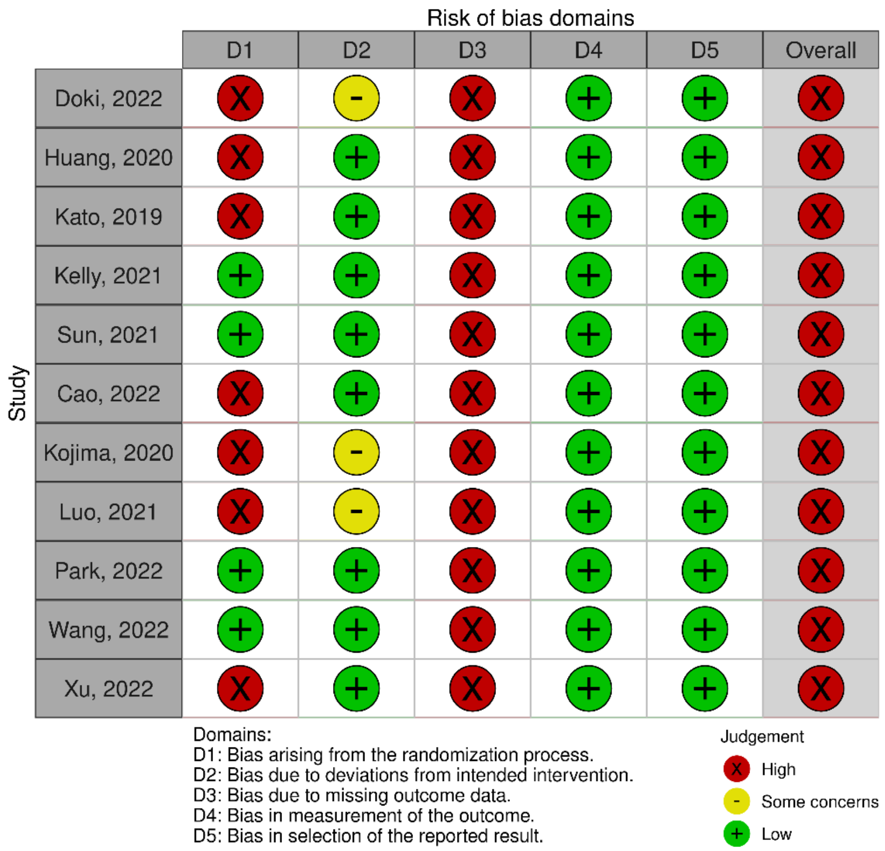

Figure S1. Quality of included trials based on Cochrane Collaboration's risk of bias assessment tool 2 (RoB 2)

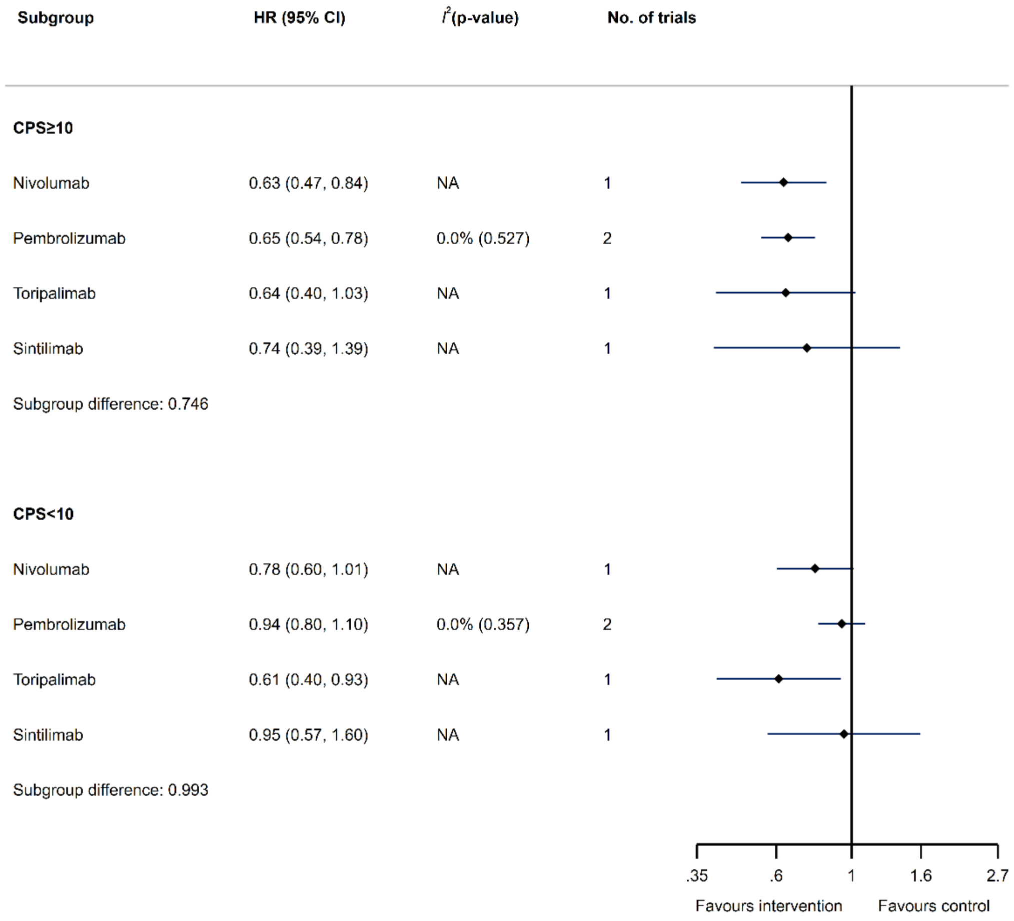

Figure S2. Forest plots of overall survival (OS) in PD-L1 positive (i.e. CPS≥10) group vs. PD-L1 negative (i.e. CPS<10) based on the type of immune checkpoint inhibitors

Subgroup	HR (95% CI)	I^2(p-value)	No. of trials
**TPS≥1**			
Drug			
Nivolumab	0.61 (0.49, 0.76)	15.9% (0.275)	2
Camrelizumab	0.59 (0.47, 0.73)	0.0% (0.941)	2
Durvalumab	0.42 (0.10, 1.76)	NA	1
Sintilimab	0.88 (0.45, 1.70)	NA	1
Subgroup difference: 0.606			
Drug target			
PD-1	0.61 (0.53, 0.70)	0.0% (0.650)	5
PD-L1	0.42 (0.10, 1.76)	NA	1
Subgroup difference: 0.640			
**TPS<1**			
Drug			
Nivolumab	0.92 (0.75, 1.12)	0.0% (0.451)	2
Camrelizumab	0.81 (0.65, 1.00)	0.0% (0.867)	2
Durvalumab	1.53 (0.48, 4.85)	NA	1
Sintilimab	0.79 (0.49, 1.28)	NA	1
Subgroup difference: 0.596			
Drug target			
PD-1	0.86 (0.75, 0.99)	0.0% (0.834)	5
PD-L1	1.53 (0.48, 4.85)	NA	1
Subgroup difference: 0.386			

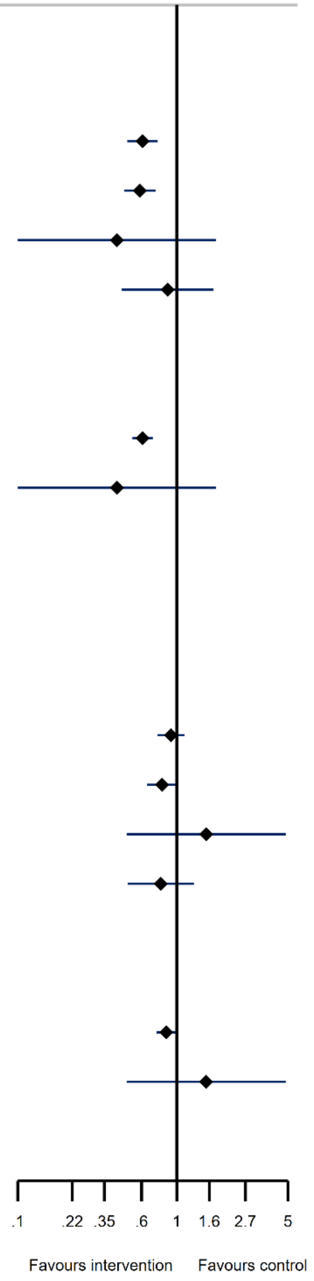

Figure S3. Forest plots of overall survival (OS) in PD-L1 positive (i.e. TPS≥1%) group vs. PD-L1 negative (i.e. TPS<1%) based on the type and target of immune checkpoint inhibitor.
